# DNA methylation‐based biomarkers of aging were slowed down in a two‐year diet and physical activity intervention trial: the DAMA study

**DOI:** 10.1111/acel.13439

**Published:** 2021-09-18

**Authors:** Giovanni Fiorito, Saverio Caini, Domenico Palli, Benedetta Bendinelli, Calogero Saieva, Ilaria Ermini, Virginia Valentini, Melania Assedi, Piera Rizzolo, Daniela Ambrogetti, Laura Ottini, Giovanna Masala

**Affiliations:** ^1^ Laboratory of Biostatistics Department of Biomedical Sciences University of Sassari Sassari Italy; ^2^ MRC‐PHE Centre for Environment 43 and Health Imperial College London London UK; ^3^ Institute for Cancer Research, Prevention and Clinical Network ‐ ISPRO Florence Italy; ^4^ Department of Molecular Medicine Sapienza University of Rome Rome Italy

**Keywords:** dietary habits, DNA methylation, epigenetic clock, epigenetic mutation load, physical activity, postmenopausal women, primary prevention trial

## Abstract

Several biomarkers of healthy aging have been proposed in recent years, including the epigenetic clocks, based on DNA methylation (DNAm) measures, which are getting increasingly accurate in predicting the individual biological age. The recently developed “next‐generation clock” DNAmGrimAge outperforms “first‐generation clocks” in predicting longevity and the onset of many age‐related pathological conditions and diseases. Additionally, the total number of stochastic epigenetic mutations (SEMs), also known as the epigenetic mutation load (EML), has been proposed as a complementary DNAm‐based biomarker of healthy aging. A fundamental biological property of epigenetic, and in particular DNAm modifications, is the potential reversibility of the effect, raising questions about the possible slowdown of epigenetic aging by modifying one's lifestyle. Here, we investigated whether improved dietary habits and increased physical activity have favorable effects on aging biomarkers in healthy postmenopausal women. The study sample consists of 219 women from the “Diet, Physical Activity, and Mammography” (DAMA) study: a 24‐month randomized factorial intervention trial with DNAm measured twice, at baseline and the end of the trial. Women who participated in the dietary intervention had a significant slowing of the DNAmGrimAge clock, whereas increasing physical activity led to a significant reduction of SEMs in crucial cancer‐related pathways. Our study provides strong evidence of a causal association between lifestyle modification and slowing down of DNAm aging biomarkers. This randomized trial elucidates the causal relationship between lifestyle and healthy aging‐related epigenetic mechanisms.

## Graphical abstract

In a 24‐month randomized factorial intervention trial, dietary and physical activity interventions slowed down DNA methylation‐based biomarkers of aging.

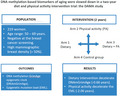

## 1 INTRODUCTION

Population aging is emerging as one of the most critical health issues, leading to medical, social, economic, and political problems. To quantify healthy aging in epidemiological and clinical studies is not straightforward. Among various biomarkers of healthy aging proposed in recent years, the epigenetic clocks, based on DNA methylation (DNAm) data, are getting increasingly accurate in predicting the individual biological age (Horvath, 2013; Horvath & Raj, 2018). The concept of epigenetic age acceleration (AA) has been introduced as the difference between predicted DNAm age and the chronological age: positive values of AA indicate unhealthy aging and *vice versa* (Horvath, 2013). Recent literature suggests epigenetic AA as a reliable biomarker of healthy aging as it has been associated with longevity (Chen et al., 2016; Dugué et al., 2018), several pathological conditions (Horvath et al., 2016), and non‐communicable disease risk factors like obesity (Horvath et al., 2014), poor physical activity (PA) (Quach et al., 2017), and low socioeconomic status (Fiorito et al., ,^2017^, ^2019^).

To date, epigenetic clocks that have gained considerable popularity in the scientific community are Horvath (Horvath, 2013) and (Hannum et al., 2013) “first‐generation clocks,” and Levine's DNAmPhenoAge (Levine et al., 2018) and Lu's DNAmGrimAge (Lu et al., 2019) “next‐generation clocks.” It has been shown that the “next‐generation clocks,” DNAmGrimAge particularly, outperform “first‐generation clocks” in predicting longevity and the onset of age‐related pathological conditions and diseases (Bergsma & Rogaeva, 2020; Lu et al., 2019). Specifically, the DNAmGrimAge is built as a linear combination of seven DNAm‐based surrogate markers of plasma proteins: adrenomedullin (ADM), beta‐2‐microglobulin (B2 M), cystatin C (Cystatin C), growth differentiation factor 15 (GDF‐15), leptin (Leptin), plasminogen activator inhibitor‐1 (PAI‐1), and tissue inhibitor metalloproteinases 1 (TIMP‐1) plus DNAm‐based biomarkers for smoking pack‐years, using DNAm values of 1,030 unique CpG sites.

Additionally, the total number of stochastic epigenetic mutations (SEMs) per individual has been proposed as an alternative biomarker of healthy aging based on whole‐genome DNAm data (Gentilini et al., 2015). The total number of SEMs per individual, also known as epigenetic mutation load (EML) (Yan et al., 2020), is defined as the sum of extreme (outliers) DNAm values per sample. Recently, (Gentilini et al., 2015) provided evidence of the exponential relationship between age and SEMs, which occurs naturally during aging as a consequence of the “epigenetic drift.” A higher EML has been associated with age‐related pathological conditions like X chromosome activation skewing (Gentilini et al., 2015) and risk factors for non‐communicable diseases like cigarette smoking, alcohol intake, exposure to toxicants, and low socioeconomic status (Curtis et al., 2019; Fiorito et al., 2019), and it is associated with increased risk of different types of cancer in prospective studies (Gagliardi et al., 2020; Wang et al., 2019). Interestingly, DNAm epigenetic clocks and EML are weakly correlated, suggesting they describe different aspects of epigenetic aging processes (Yan et al., 2020).

A fundamental property of epigenetic is the potential reversibility of the effect, raising questions about the possible slowdown of epigenetic aging by improving lifestyle. Recent observational studies provided evidence that smoking‐related DNAm modifications tend to reverse after smoking cessation in a time‐dependent manner (Guida et al., 2015), and epigenetic AA due to early‐life social adversities can be partially reversed improving lifestyle and social conditions in adulthood (Fiorito et al., 2017). A pilot clinical trial conducted on nine volunteers suggests that the epigenetic clock could be reversed after one‐year treatment with a cocktail of drugs based on recombinant human growth hormone (Fahy et al., 2019).

In this study, we aimed to investigate whether modifying dietary habits and increasing PA have favorable effects on biological aging, measured using both the DNAmGrimAge and the EML, in healthy postmenopausal women. This study sample consists of 219 adult post‐menopausal women from the “Diet, Physical Activity, and Mammography” (DAMA) study: a single‐center, 24‐month randomized intervention trial whose primary aim was to investigate whether mammographic breast density (an established independent risk factor for breast cancer development) could be reduced in healthy postmenopausal women by modifying their dietary habits and physical activity levels (Masala et al., 2019).

## 2 RESULTS

After DNAm data quality controls and filtering, this study sample include 219 DAMA participants, distributed into four trial study arms (*arm 1*: dietary intervention, *arm 2*: PA intervention, *arm 3*: dietary +PA intervention, and *arm 4*: control group), with whole‐genome DNAm measured from blood collected at baseline and after two years of intervention. For each sample, we computed the total number of SEMs and DNAmGrimAge measures. For statistical comparisons, we used a logarithm transformation of the total number of SEMs (referred to as EML henceforth), and DNAmGrimAge Acceleration (referred to as DNAmGrimAA henceforth) was defined as the residuals of the regression of DNAmGrimAge on chronological age as described by Lu and colleagues (Lu et al., 2019).

### 2.1 Association analyses at baseline

In Table S1, we reported the characteristics of the study sample by study arm at baseline. There were no statistically significant differences among the four groups considering anthropometric and lifestyle characteristics, nor DNAmGrimAA, whereas the EML differed by groups at baseline (ANOVA test p‐value =0.01).

In Table 1, we reported the results of two multivariate linear regression models having either baseline DNAmGrimAA or EML used as the outcome, and baseline anthropometric and lifestyle characteristics entered as the predictors. DNAmGrimAA was associated with obesity (β = 0.80 95% CI 0.11–1.49, *p* = 0.02 comparing overweight with normal‐weight; β = 2.53 95% CI 1.28–3.78, *p* = 0.0001 comparing obese with normal‐weight) and smoking (β = 0.88 95% CI 0.23–1.52, *p* = 0.01 comparing former with never smokers) adjusting for the other risk factors in Table 1, whereas EML was not associated with any lifestyle variables at baseline.

**TABLE 1:** Associations of biological aging measures with anthropometric and lifestyle variables at baseline: estimates, 95% confidence intervals, and p‐values were derived from multivariate linear regression models. The effect of each baseline characteristic on DNAmGrimAA and EML is adjusted for all the other covariates in the table

	DNAmGrimAA		EML	
	Estimate (95% CI)	p	Estimate (95% CI)	p
BMI (ref. <25)	‐	‐	‐	‐
25–30	0.80 (0.11; 1.49)	0.02	0.23 (−0.06; 0.52)	0.14
>30	2.53 (1.28; 3.78)	0.0001	−0.10 (−0.65; 0.45)	0.73
Smoking (ref. Never)	‐	‐	‐	‐
Former	0.88 (0.23; 1.52)	0.01	−0.17 (−0.44; 0.10)	0.10
Education (ref. Primary)	‐	‐	‐	‐
secondary	0.08 (−0.70; 0.86)	0.85	−0.05 (−0.38; 0.28)	0.78
University or above	0.47 (−0.37; 1.31)	0.27	−0.01 (−0.36; 0.34)	0.94
Physical Activity (ref. Inactive)	‐	‐	‐	‐
Mod. Inactive	0.00 (−0.84; 0.84)	0.99	−0.13 (−0.48; 0.22)	0.47
Mod. Active	−0.79 (−1.69; 0.11)	0.09	−0.06 (−0.43; 0.31)	0.75
Active	−0.14 (−1.20; 0.92)	0.80	0.10 (−0.35; 0.55)	0.66
Coffee (ref. <= 3 cups/day)	‐	‐	‐	‐
> 3 cups/day	−0.26 (−0.91; 0.39)	0.43	0.08 (−0.19; 0.35)	0.57
Alcohol (ref. Never)	‐	‐	‐	‐
<= 1 drink/day	0.14 (−0.74; 1.02)	0.75	0.04 (−0.33; 0.41)	0.83
> 1 drink/day	0.85 (−0.23; 1.93)	0.12	0.30 (−0.15; 0.75)	0.20
Dietary style (ref. good)	‐	‐	‐	‐
bad	0.43 (−0.22; 1.08)	0.19	−0.07 (−0.34; 0.20)	0.64
Breastfeeding (ref. <= 3 months)	‐	‐	‐	‐
> 3 months	0.11 (−0.54; 0.76)	0.73	−0.17 (−0.44; 0.10)	0.23
Oral contraceptives (ref. Never)	‐	‐	‐	‐
Ever	−0.11 (−0.76; 0.54)	0.74	0.22 (−0.05; 0.49)	0.11
Menopausal hormones (ref. Never)	‐	‐	‐	‐
Ever	0.02 (−0.69; 0.73)	0.95	−0.16 (−0.45; 0.13)	0.30

Table 2 reports Pearson correlation coefficients (and corresponding p‐values) among the two epigenetic aging biomarkers and dietary variables at baseline. Higher consumption of fruit and vegetables was associated with decreased DNAmGrimAA (*p* = 0.05 and *p* = 0.002, respectively), whereas a higher consumption of processed meat was associated with increased EML (*p* = 0.01).

**TABLE 2:** Pearson correlation test comparing biological aging measures with dietary variables at baseline.

Dietary variables (gr/die)	DNAmGrimAA		EML	
	Pearson R	p	Pearson R	p
Vegetables	−0.14	0.05	0.14	0.06
Fruit	−0.21	0.001	0.09	0.19
Red meat	0.08	0.26	0.07	0.29
Processed meat	−0.05	0.48	0.18	0.01
Poultry	0.01	0.84	−0.02	0.72
Fish	0.07	0.30	0.13	0.06
Dairy products	−0.08	0.26	0.02	0.78
Kcal	−0.07	0.27	0.10	0.13

### 2.2 Association analyses after the intervention

**FIGURE 1:**
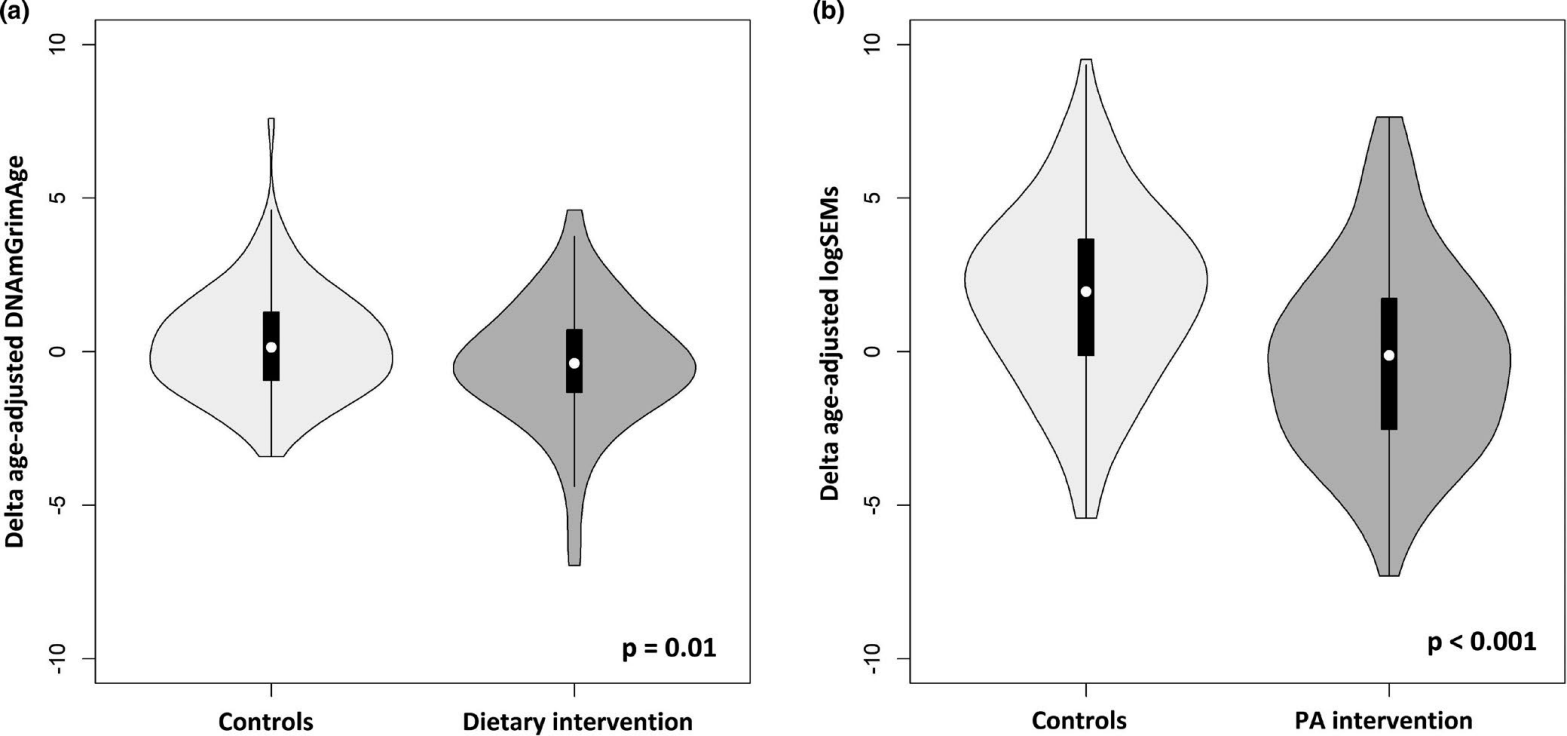
Violin Plots: (a) Distribution of the delta DNAmGrimAA (DNAmGrimAA after two‐year trial minus DNAmGrimAA at baseline) in women participating in the dietary intervention vs. controls. Dietary intervention leads to a significant reduction of the delta DNAmGrimAA (0.66 years), computed via linear regression model adjusted for anthropometric and lifestyle characteristics at baseline. (b) Distribution of the delta age‐adjusted EML (EML after two‐year trial minus EML at baseline) in women participating in the PA intervention vs. controls. PA intervention leads to a significant reduction of the delta age‐adjusted EML (2 years), computed via linear regression model adjusted for anthropometric and lifestyle characteristics at baseline

We run a difference‐in‐difference model to estimate the differential changes of DNAmGrimAA and EML in the treated group compared with the control group during the two‐year intervention. We applied a two‐step approach: First, we defined the “delta DNAmGrimAA” and “delta EML” as the difference between the two epigenetic aging biomarkers measured after and before the intervention. In Figure 1, we reported the distribution of delta DNAmGrimAA (Figure 1a) and delta EML (Figure 1b) in controls and intervention groups (dietary intervention for DNAmGrimAA and PA intervention for EML). The average delta DNAmGrimAA were 0.25 (95% CI −0.07 to 0.57) and −0.41 (95% CI −0.79 to −0.03) in the control and dietary intervention groups, respectively. The average delta EML were 1.82 (95% CI 1.28 to 2.37) and −0.23 (95% CI −0.82 to −0.35) in the control and PA intervention groups, respectively.

Then, we have estimated the differential changes of DNAmGrimAA and EML through linear regression models using the delta DNAmGrimAA and delta EML as the outcomes (control group as the reference). The dietary intervention led to a significant reduction of delta DNAmGrimAA (β = −0.66, 95% CI −1.15 to −0.17, *p* = 0.01, Table 3), whereas the PA intervention caused a significant reduction of the delta EML (β = −2.06, 95% CI −2.84 to −1.28, *p* < 0.0001, Table 3). There was no significant reduction of DNAmGrimAA associated with the PA intervention nor reduced EML associated with the dietary intervention (Table 3). For both DNAmGrimAA and EML, the estimated differences presented in Table 3 (i.e., the β coefficients) can be interpreted as the change in biological age (in years) compared with the reference group (see Methods for more details).

**TABLE 3:** Average differences and 95% confidence intervals (CIs) of DNAmGrimAA and EML measured before the randomized trial minus DNAmGrimAA and EML measured after the randomized trial (first two columns); and differential changes in the delta DNAm‐based aging measures (difference‐in‐difference model, third column). Comparison of the dietary intervention (arms 2 and 4) with the control group (arms 1 and 3) on the top of the table; comparison of the PA intervention (arms 1 and 4) with the control group (arms 2 and 3) on the bottom of the table. Estimates, 95% Cis, and p‐values come from a two‐step difference‐in‐difference model

	Mean (95% CI) difference (measure after intervention minus measure before the intervention) in control group (arms 2 and 4)	Mean (95% CI) difference (measure after the intervention minus measure before the intervention) in dietary intervention group (arms 1 and 3)	Differential effect of dietary intervention vs. control group	
			Estimate (95% CI)	p
*DNAmGrimAA*	0.25 (−0.07, 0.57)	−0.41 (−0.79, −0.03)	−0.66 (−1.15, −0.17)	0.01*
*EML*	1.00 (0.41, 1.60)	0.63 (0.03, 1.23)	−0.37 (−1.21, 0.48)	0.39

	Mean (95% CI) difference (measure after the intervention minus measure before the intervention) in control group (arms 1 and 4)	Mean (95% CI) difference (measure after the intervention minus measure before the intervention) in PA intervention group (arms 2 and 3)	Differential effect of PA intervention vs. control group	
			Estimate (95% CI)	p
*DNAmGrimAA*	−0.12 (−0.43, 0.20)	−0.03 (−0.43, 0.37)	0.09 (−0.42, 0.60)	0.73
*EML*	1.82 (1.28, 2.37)	−0.23 (−0.82, 0.36)	−2.06 (−2.84, −1.28)	<0.001*

### 2.3 Additional investigation on the eight DNAmGrimAA components

**FIGURE 2:**
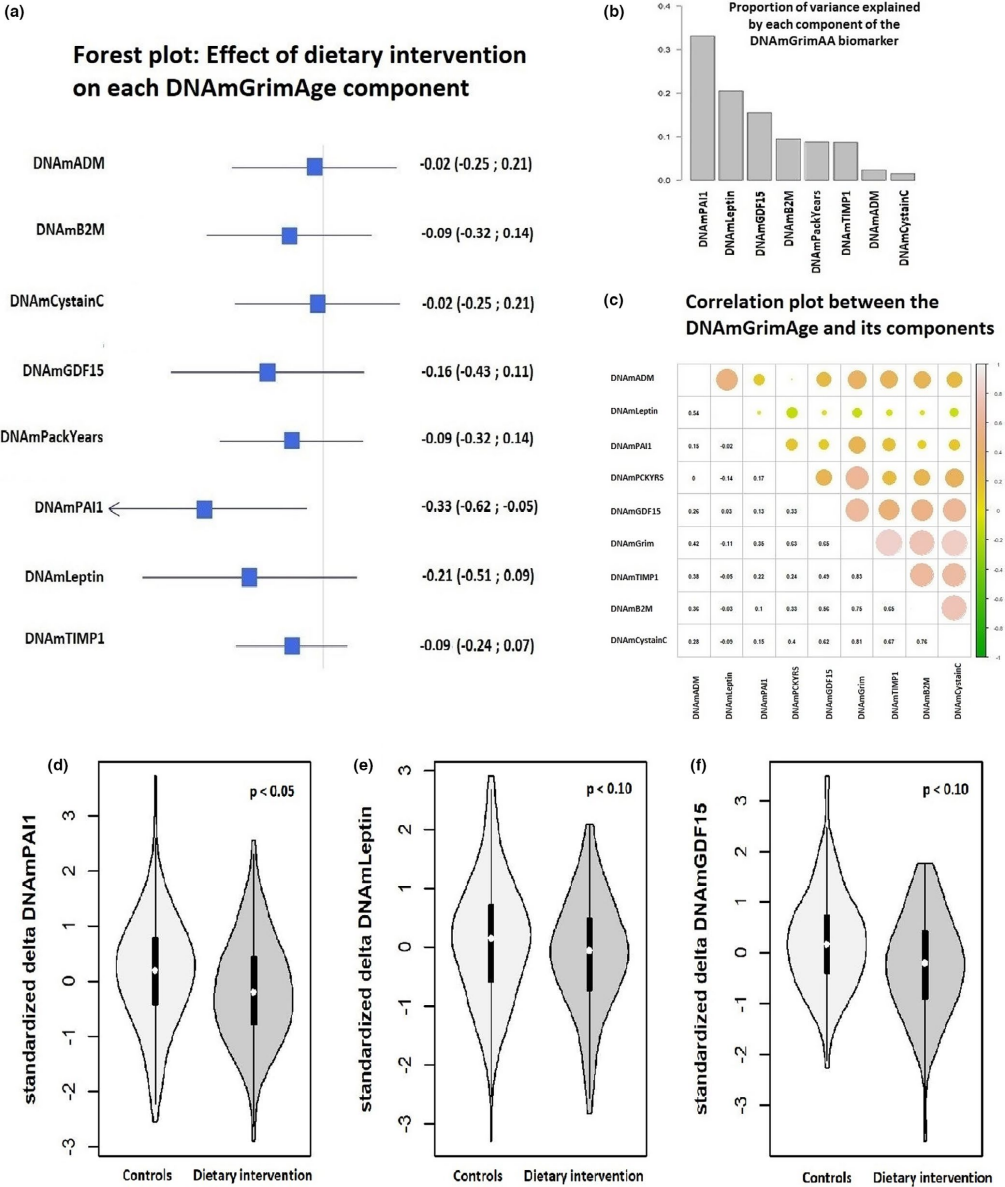
Analysis of the eight components of the DNAmGrimAge: a. Forest plot indicating the effect of the dietary intervention on each component of the DNAmGrimAge (expressed as standard deviations change to be comparable among them). b. Proportion of variability explained by each component of the DNAmGrimAge. c. Correlation matrix among DNAmGrimAge and its components. d‐e‐f. Violin plots. Distribution of the standardized delta DNAmPAI1, delta DNAmLeptin, and delta DNAmGDF15 (measure after two‐year trial minus measure at baseline) in women participating in the dietary intervention vs. control group

**FIGURE 3:**
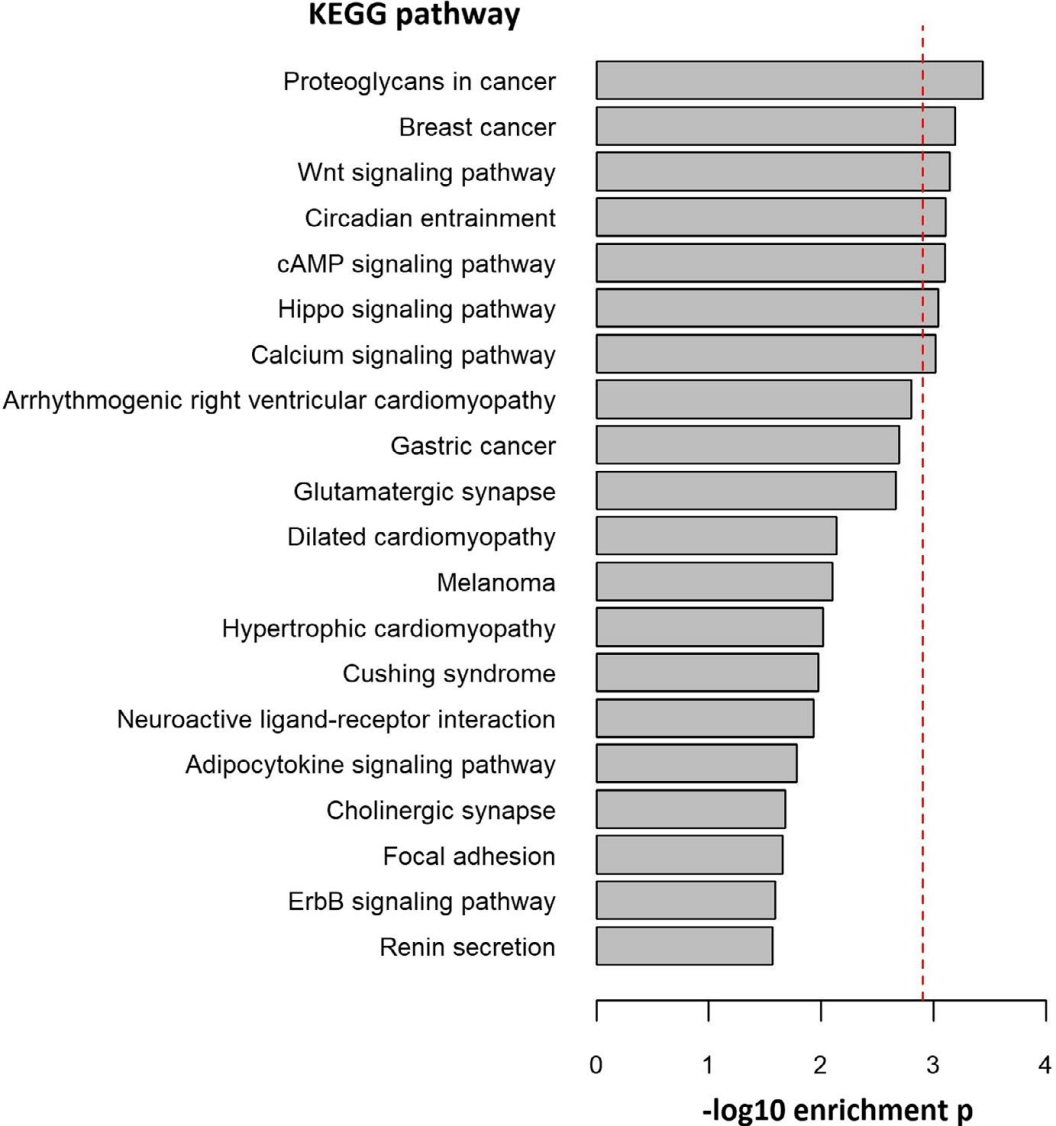
PArSEMs CpGs gene ontology enrichment analysis. Top 20 KEGG pathways and ‐log10 enrichment p‐values. Red dotted line indicates the FDR threshold of significance. After FDR correction for multiple testing, PArSEMs were significantly enriched in seven KEGG biological pathways

We further investigate the effect of dietary intervention separately on each component of the DNAmGrimAA in order to identify which contributed the most on the previously described association. The results of the single‐component analyses are summarized in Figure 2a. DNAmPAI1 biomarker was the only DNAmGrimAA component with a significant reduction after the two‐year dietary intervention (β = −0.33 standard deviations, 95% CI −0.62 to −0.05, comparing women who participated in the dietary intervention vs. controls, Figure 2b) and contribute for more than 30% of the explained variability (Figure 2c). Also, reduction of DNAmLeptin and DNAmGDF15 provided a substantial contribution (20% and 15% respectively, Figures 2, 3).

### 2.4 Enrichment analyses on epimutated CpG sites

We further investigated the biomolecular pathways involved in the reduction of EML caused by the PA intervention and the stability of SEMs over these two years. The majority of the identified CpG sites carrying a SEM at baseline (i.e., before the intervention) were still epimutated after the two‐year trial (the average proportion of “stable” SEMs per individual was 69%, ranging from 54% to 89%). We performed additional investigation of what we named “physical activity‐related reversible SEMs” (PArSEMs), that is, those CpG sites in which we found a SEM at baseline but not after the PA intervention trial.

PArSEMs were enriched in non‐CpG islands (*p* = 0.02, Table S3), genomic regions characterized by heterochromatin/low transcriptional signal/copy number variants (*p* < 0.0001, Table S4), and transcription factor binding sites (TFBS) of EZH2 and SUZ12 (*p* = 0.001 and *p*=0.006, respectively, Table S5). Furthermore, the top 20 KEGG pathways from the gene ontology enrichment analysis are listed in Figure 3. After false discovery rate (FDR) correction for multiple testing, PArSEMs were enriched in the following seven KEGG pathways: *hsa05205* (“Proteoglycans in cancer,” *p* = 0.0004), *hsa05224* (“Breast cancer,” *p* = 0.0006), *hsa04310* (“Wnt signaling pathway,” *p* = 0.0007), *hsa04713* (“Circadian entertainment,” *p* = 0.0008), *hsa04024* (“cAMP signaling pathway,” *p* = 0.0008), *hsa04390* (“Hippo signaling pathway,” *p* = 0.0009), and *hsa04020* (“Calcium signaling pathway,” *p* = 0.001) (Table S5).

### 2.5 Sensitivity analyses

For sensitivity analyses, we repeated the previously described linear regression models including additional adjustment for estimated white blood cells (WBC) proportions. The sensitivity analyses confirmed the reduction of the delta DNAmGrimAA in women participating in the dietary intervention (β = −0.42 95% CI −0.83 to −0.01, *p* = 0.05) and the reduction of delta EML in women participating in the PA intervention (β = −2.04 95% CI −2.82 to −1.26, *p* = 0.001).

## 3 DISCUSSION

Various cross‐sectional studies provided evidence of a favorable effect of a healthy lifestyle on several biological aging indicators, including DNAm‐based aging biomarkers (Koop et al., 2020; Quach et al., 2017). However, longitudinal and intervention studies are needed to clarify causality and accurately quantify the benefit of improving lifestyle at a biomolecular level. A recent review by ElGendy and colleagues summarizes the effect of several intervention studies of folic acid and B vitamins supplementation on whole‐genome DNAm profiles. They conclude that the effects on DNAm are gene and site‐specific, depending on cell type and tissue, and the duration of the intervention, making the results difficult to interpret. However, they observed consensus on increased global DNAm consequence of folic acid and B vitamins supplementation (Elgendy et al., 2018). DNAm‐based biological aging measures provide an easily interpretable summary measure of the state of health of an individual and can be used to investigate the beneficial effects of improving lifestyle on aging‐related epigenetic mechanisms (Lu et al., 2019).

Few intervention studies have evaluated the effect of improving dietary habits on DNAm epigenetic clocks at the current stage, whereas no studies investigated changes in the EML. For example, Sae‐Lee and colleagues investigated folic acid and vitamin B12 supplementation in a randomized trial including 44 participants (+13 participants from a non‐randomized trial), concluding that the slowing down of the DNAm aging is gender‐ and *MTHFR* genotype‐specific (Sae‐Lee et al., 2018). More recently, Fitzgerald and colleagues provide evidence of reversal epigenetic clocks improving diet and lifestyle in a randomized trial including 38 participants (Fitzgerald et al., 2021). Although there is strong evidence that physical exercise has favorable effects on epigenetic mechanisms (Ferioli et al., 2019), there is a lack of intervention studies on increasing physical activity associated with longitudinal measures of epigenetic clocks and aging‐related epigenetic drift.

In this study, we explored the effects of a two‐year dietary and PA intervention trial on two DNAm‐based biomarkers of biological aging: DNAmGrimAge, since it has been shown it outperforms other epigenetic clocks in predicting aging‐related outcomes (McCrory et al., 2020), and the epigenetic mutation load (EML, as a biomarker of the aging‐related epigenetic drift), in more than 200 healthy postmenopausal women from the DAMA study.

Our results on the analyses performed at baseline (before the intervention trial) confirmed previously observed cross‐sectional associations between the epigenetic clocks and risk factors for non‐communicable diseases, like obesity, consumption of processed meat (with unfavorable effects), and consumption of fruit and vegetables (with favorable impacts). Interestingly, the two epigenetic biomarkers of aging likely describe different aspects of the aging‐related molecular mechanisms, as they are associated with different risk factors for non‐communicable diseases. In fact, in our sample, obesity is associated with DNAmGrimAA but not with EML. Similarly, higher DNAmGrimAA is associated with lower consumption of fruit but not with higher consumption of red meat, whereas an inverse pattern of associations was observed for the EML.

### 3.1 Dietary improvement slows down the DNAmGrimAge biomarker

The main aim of the present study was to compare the DNAm‐based aging biomarkers before and after the intervention. Our results highlighted a significant slowing down of the epigenetic aging processes because of the improved dietary quality and increased PA. Specifically, the dietary intervention led to a significant slowing down of the DNAmGrimAA biomarker, whereas the PA intervention had a significant effect on the total number of SEMs.

We performed additional statistical analyses to identify which components of the DNAmGrimAA contributed most to the observed association. The results highlighted a significant decrease of the DNAm surrogate measure of the plasma protein PAI‐1 as well a substantial decrease of Leptin and GDF‐15 proteins DNAm surrogates among women in the dietary intervention trial arm. Our results provide further support on the association of specific plasma proteins with healthy aging and longevity. High level of PAI‐1 has been associated with a number of age‐related conditions and lifespan (Khan et al., 2017), and previous studies reported a beneficial effect of PA on PAI‐1 serum levels (Lira et al., 2010). Further, DNAm surrogate for PAI1 has a strong association with metabolic syndrome, obesity, and fatty liver (Lu et al., 2019). Leptin is mainly produced in the white adipose tissue and is one of the main catabolic regulators of food intake and energy expenditure. During aging, a significant increase in Leptin resistance leads to unfavorable health outcomes (Balaskó et al., 2014). Evidence indicates that caloric restriction reduces Leptin levels in a dose–response manner (Hong et al., 2018). Finally, the dietary intervention was associated with decreased levels of GDF‐15 protein DNAm surrogate, that emerged recently as a biomarker of inflammation, regulation of apoptosis, cell repair, healthy aging, and a robust prognostic protein in patients with different diseases such as heart diseases and cancer (Baek & Eling, 2019; Luan et al., 2019).

### 3.2 Increasing physical activity slows down the EML biomarker

Increasing evidence indicates that aging is associated with an accumulation of SEMs, and in turn, the total number of SEMs is associated with an increased risk for several cancer types (Gentilini et al., 2015, 2017; Wang et al., 2019). We observed a slowing down of the EML biomarker in women who participated in the PA intervention trial compared with the control sample. Such slowing down of the EML caused by increased PA may be explained with an adaptive increase in antioxidant capacity and reduction of reactive oxygen species (ROS), which in turn leads to a higher DNA repair capacity and therefore a lower number of dangerous SEMs (Grazioli et al., 2017; Kietzmann et al., 2017).

Although a large proportion of the identified SEMs was stable over time, many SEMs were no longer present after the PA intervention. A more‐in‐depth investigation indicates that reversible SEMs are enriched in non‐CpG islands and genomic regions characterized by heterochromatin, low transcriptional signal, and copy number variants. These results are in line with previous observations of lower DNAm variability in CpG islands and CpG‐rich genomic regions (Palumbo et al., 2018). Functional characterization of PArSEMs CpGs highlights an enrichment of reversible epimutations in TFBS of two members of the Polycomb Repressive Complex 2 (PRC2) proteins: EZH2 and SUZ12. Interestingly, previous studies indicate that a lower number of SEMs in genes targeted by these two proteins, mostly tumor suppressor genes, is associated with a lower risk of future cancer development (Gagliardi et al., 2020). Additionally, KEGG pathway gene ontology enrichment analysis shows that PArSEMs are enriched in several cancer‐related pathways such as hsa05205 (“Proteoglycans in cancer”), hsa05224 (“Breast cancer”), hsa04310 (“Wnt signaling pathway”), and hsa04024 (“cAMP signaling pathway”). Thus, the established association between increasing PA and reduced cancer risk might be partly explained via a reduction of epimutations in critical cancer‐related pathways.

## 4 CONCLUSIONS

We provided strong evidence of a causal association of improving dietary habits and increasing physical activity on DNAm‐based biomarkers of healthy aging. It is worthy to note that DAMA study is intentionally based on non‐extreme interventions, meaning that relatively easily achievable changes in one's lifestyle behaviors lead to a significant slowing down of biological aging biomarkers, which in turn are associated with higher longevity, lower risk of developing age‐related diseases, and increased quality of life in the older age. Further, our results indicate that dietary quality and physical activity influence epigenetic aging through complementary molecular mechanisms, suggesting that their effect is potentially cumulative rather than interchangeable. In conclusion, our results provide further evidence about the importance of policy intervention programs to promote a healthy diet and physical activity, leading to a substantial reduction of the burden for many aging‐related pathological conditions and diseases. Additionally, our results provide a step forward in understanding the biological mechanism of aging and identifying health‐related biomarkers.

### 4.1 Strength and limitations

Since this was a secondary analysis, the relatively modest sample size is a possible limitation of this study. The original factorial study design included four arms (*arm 1*: diet, *arm 2*: PA, *arm 3*: diet +PA, and *arm 4*: controls), but for statistical comparisons, we used the two main intervention groups (arms 1 and 3 for investigating the effect of dietary intervention, and arms 2 and 3 for investigating the effect of PA intervention). However, a post hoc power analysis of the study indicates that our analytical strategy makes this study well‐powered (β > 0.80) considering the effect sizes observed in linear regressions. On the contrary, the factorial design of the DAMA study and our analytical choice make that, in estimating the effect of the dietary intervention, around 50% of the treated group and around 50% of the controls have completed the physical activity intervention also (and vice versa considering the effect of PA intervention), leading to possible confounding of the results. This study includes only women making impossible to investigate possible differential effect by gender. Finally, due to the limited sample size, we were not able to include extra stratified statistical analyses to test additional hypotheses (e.g., whether the effect of the trial is higher among obese women at baseline), underlining the need for further investigations in the field.

This study also has several strengths. To the best of our knowledge, this is the first study investigating longitudinal changes in the DNAmGrimAge and epigenetic mutation load with a suitable lifestyle intervention, allowing a robust causal interpretation of the results. Of note, our results are not biased by the presence of pathological conditions or smoking (well known for having a strong influence on DNAm profiles and epigenetic aging) since the study sample is composed of healthy non‐smokers women.

## 5 EXPERIMENTAL PROCEDURES

### 5.1 Study sample

The DAMA study was a single‐center, 24‐month randomized intervention trial (Trial Registration ID: ISRCTN28492718) with a 2x2 factorial design, whose primary aim was to investigate whether mammographic breast density (MBD) could be reduced in high‐MBD (>50%) healthy post‐menopausal women by modifying their dietary habits and/or PA levels (Masala et al., 2019). Study participants were selected in 2009–2010 among postmenopausal women aged 50–69 years that attended the local breast cancer screening program in Florence, Italy (Masala et al., 2014). Women were eligible for inclusion if they had a negative screening mammogram with MBD >50% (assessed using the BI‐RADS classification (Liberman and Menell, 2002)); those selected for a second‐stage diagnostic procedure following the screening mammogram were excluded regardless of the final outcome of the diagnostic process. Other exclusion criteria were as follows: recent (past 12 months) hormone replacement therapy use; current smoking, or having quit smoking by <6 months; being previously diagnosed with cancer (except non‐melanoma skin cancer) or suffering from any illness that could hamper an active participation in the study activities.

At baseline, all participants provided information on dietary habits and lifestyle (including household, occupational and leisure‐time PA) by filling two questionnaires previously used within the EPIC (European Prospective Investigation into Cancer and Nutrition) study (Palli et al., 2003), and had their anthropometric measures taken using standardized procedures. A fasting venous blood sample was taken, divided into plasma, red cells, and buffy‐coat aliquots, and stored together with urine samples in the project biobank. Each woman was then randomly assigned to one of the four study arms (diet, PA, diet+PA, and control) according to a permuted‐block randomization scheme stratified by age (50–59 vs. 60–69 years) and body mass index (BMI) category (<25 vs. ≥25 kg/m^2^), with a constant block size (n=4).

Study participants assigned to the dietary intervention (*arm 1*) were counseled to adopt a diet based on the consumption of plant foods, with a low glycemic load, low in saturated‐ and *trans*‐fats and alcohol, and rich in antioxidants. The change in dietary habits was aimed to be achieved in an isocaloric context, as no advice was given about the quantity of food to be consumed. The intervention objectives included: (a) replacement of refined grains with whole grains; (b) consumption of at least one portion of raw vegetables and one portion of cooked vegetables at each meal; (c) consumption of fish at least 2–3 times weekly; (d) reduction of the consumption of fresh and processed red meat to less than once weekly; (e) consumption of at least 3–4 portions of legumes and pulses per week; (f) daily consumption of at least 2–3 portions of fruit; (g) cakes and desserts consumed no more than once weekly; (h) no more than 1 portion/day of milk or yogurt and 2 portions/week of cheese; (i) exclusive use of extra‐virgin olive oil as dressing and cooking fat; and (j) consumption of no more than one glass of wine daily at meals for those already used to drink alcohol. In addition, women allocated to the dietary intervention study arm were also requested to attend six group meetings and eight cooking classes over the course of the study.

Women randomized to the PA intervention (*arm 2*) were asked to increase their moderate‐level recreational PA up to 1 hour/day (corresponding to about 3 MET‐hours day [MET=metabolic equivalent]), to be combined with a more strenuous activity accounting for 6–10 MET‐hours weekly. Women were also requested to attend weekly a one‐hour session led by trained PA experts in an appropriate fitness facility and were provided with some equipment for home exercises. Finally, the study protocol also included participation in six group sessions and six collective walks supervised by the study team.

Women assigned to the diet +PA intervention (*arm 3*) were requested to change both their dietary habits and PA levels by combining the protocols of arms 1 and 2.

Study participants assigned to any intervention arm (1, 2, or 3) were requested to keep five written one‐week diaries on diet and/or PA levels (depending on study arm), which were then reviewed by the study personnel to monitor the achievement of the study objectives and provide further tailored counseling to the participants.

Women randomly assigned to the control group (arm 4) received general advice on healthy diet and PA levels according to the recommendations from the World Cancer Research Fund (WCRF) report 2007 (Wiseman, 2008), were invited to attend a group meeting taking place in the first six months of the study, and were distributed *ad hoc* printed material.

At the end of the study (24±3 months from enrollment, coinciding with the time of the next mammographic screening), all study participants underwent a final visit, in which the same protocol as at the baseline visit was applied.

Compliance with the proposed interventions was good, with an increased consumption of vegetables and legumes, and a reduced consumption of meat and cakes, observed women assigned to the dietary intervention group, and an increase in all type of recreational physical activity for those allocated to the PA group (Masala, 2019).

In the main analysis, a decrease in MBD was observed among women in the dietary intervention and in the PA group compared to controls, while no significant effect on MBD was found among women in the double intervention group (Masala, 2019).

### 5.2 Genome‐wide DNA methylation analyses

Buffy coats stored in liquid nitrogen were thawed, and genomic DNA was extracted using the ReliaPrep Blood gDNA Miniprep System Kit (Promega). The concentration of the genomic DNA was assessed by Qubit fluorimetric quantitation (Thermo Fisher Scientific). 500 ng of DNA was bisulfite‐converted using the EZ‐96 DNA Methylation‐Gold Kit (Zymo Research) and hybridized to Illumina Infinium HumanMethylation450 BeadChips (Illumina). Matched pairs (pre‐ and post‐intervention) were arranged randomly on the same array. All the chips were subsequently scanned using the Illumina HiScanSQ system. Control probes included in the microarray were used to assess bisulfite conversion efficiency and to exclude lower‐quality samples from further analyses.

### 5.3 Statistical analyses

#### 5.3.1 Data pre‐processing

Initial dataset has DNAm data for 482,421 CpG sites in 448 samples (224 matched pairs, pre‐/post‐intervention): 57 women in *arm 1* (diet); 56 women in *arm 2* (PA); 53 women in *arm 3* (diet +PA); and 58 women in *arm 4* (controls). Five samples were discarded for low bisulfite conversion total intensities according to the Illumina guidelines (Figure S1), leading to a final study sample of 219 matched pairs (pre‐ and post‐intervention). Potentially, cross‐hybridizing probes and those containing SNPs with minor allele frequency lower than 0.05 in European were excluded from the analysis (McCartney et al., 2016). Probes on Y chromosome and those with non‐unimodal distribution were also excluded, as well as those with low call rate (lower than 95%). The final dataset has 343,439 probes in 438 (219 pairs) samples. Differences by batch for fluorescence intensities of methylated and non‐methylated probes were removed using ComBat algorithm (Müller et al., 2016) (Figure S2). The proportion of WBC per sample was computed according to Houseman algorithm (Houseman et al., 2012).

#### 5.3.2 Computation of DNAmGrimAge

We computed the epigenetic age acceleration (AA) measures according to the algorithm described by Lu et al. (Lu et al., 2019). Briefly, DNAmGrimAge is computed in two steps: (1) computation of DNAm surrogate of seven plasma proteins and smoked pack‐years, using a total of 1,030 CpGs; (2) computation of the DNAmGrimAge as a linear combination of the eight DNAm surrogates plus chronological age and sex. Weights were defined using a penalized regression model (Elastic‐net regularization). DNAmGrimAge acceleration (DNAmGrimAA) is defined as the residuals of the regression of epigenetic on chronological age. Since DNAmGrimAge may be correlated with WBC proportions, the DNAmGrimAA WBC‐adjusted is defined as the residuals from the linear regression of DNAmGrimAA on WBC percentage. We used DNAmGrimAA for the analyses presented in the main text, and the measure adjusted for WBC for sensitivity analyses.

#### 5.3.3 Identification of stochastic epigenetic mutations (SEMs)

We identified SEMs based on the procedure described by Gentilini et al. (Gentilini et al., 2015). Specifically, for each CpG, considering the distribution of DNA methylation beta values across all samples, we computed the interquartile range (IQR)—the difference between the 3rd quartile (Q3) and the 1st quartile (Q1)—and we defined a SEM as a methylation value lower than Q1‐(3×IQR) or higher than Q3+(3×IQR). Finally, for each individual, we computed the total number of SEMs across the assay. Since the number of SEMs increased exponentially with age, we used a logarithmic transformation of the total number of SEMs (named EML) for all association analyses.

#### 5.3.4 Regression models at baseline

We investigated the association of DNAmGrimAA and EML at baseline with lifestyle and anthropometric characteristics at baseline via multivariate linear regression models. We used the epigenetic aging biomarkers as the outcomes; BMI, smoking habits (former/never), education, coffee and alcohol intake, PA, and dietary quality as the predictors. Dietary quality was defined using the Mediterranean diet score (bad diet =MDS <5; good diet =MDS ≥5) (Fasanelli et al., 2019).

#### 5.3.5 Difference‐in‐difference models

To investigate whether the two years dietary and PA intervention had positive effects on (meaning a reduction of) the two biological aging biomarkers, we ran linear regression models. We used the delta DNAm‐based biomarker (epigenetic measure after the two‐year trial minus those at baseline) as the outcomes and intervention group as the predictor (control group as the reference). In order to make the effect sizes of the EML biomarker comparable with those of DNAmGrimAA (i.e., expressed as years of increasing biological age), we re‐scaled both the effect sizes and the standard deviation of the EML by a factor σ = σ_AA_ / σ_SEMs_, where σ_AA_ is the deviation of DNAmGrimAA and σ_SEMs_ is the standard deviation of the EML variable. After the linear transformation, the scaled effect size of EML can be interpreted as years of increasing biological age, as is the case of DNAmGrimAge.

To investigate which of the eight components of the DNAmGrimAA have the highest contribution in the observed associations, we repeated the analyses using each component as the outcome, separately. To make the effect sizes comparable among them, estimates from the regression models were expressed as standard deviations increase.

In all the regression models, comparisons with p‐value lower than 0.05 were considered statistically significant.

#### 5.3.6 Enrichment analyses

The genomic locations of SEMs were annotated by merging the Illumina information on the chromosomal position of each probe with ENCODE/NIH Roadmap Chromatin ImmunoPrecipitation Sequencing (ChIP‐Seq) data for chromatin states and transcription factor binding sites (TFBS) in untreated human embryonic stem cells (hESC) (ENCODE Project Consortium, 2012). We investigated whether SEMs were enriched in functional genomic regions using the procedure implemented in the R package regioneR (Gel et al., 2016). Briefly, the algorithm is specifically designed to test whether a set of genomic loci significantly overlap with a set of genomic regions, using a permutation procedure that controls the type I error rate and avoids spurious associations driven by the intrinsic structure of the DNA (i.e., relationship between CG content, gene promoters, and copy number alterations) (Gel et al., 2016).

We investigated enrichment of SEMs according to (1) the “relation to CpG islands” as defined in the Illumina annotation file, which has four mutually exclusive categories: CpG island, Shelf, Shore, non‐CpG island (or “open sea” region) plus evidence for “Open chromatin state” and evidence for “DNase hypersensitivity” according to the Illumina annotation file (information from the ENCODE project (Dunham, 2012)); (2) the chromatin state in human embryonic stem cells (hESC) according to the ENCODE ChIP‐Seq experiments (Dunham, 2012), with 11 mutually exclusive categories: “active promoter,” “weak promoter,” “inactive/poised promoter,” “strong enhancer,” “weak/poised enhancer,” “insulator,” “transcriptional transition/elongation,” “weak transcribed,” “Polycomb‐repressed,” “heterochromatin/low signal/copy number variation (CNV),” and “non‐regulatory regions”; (3) the TFBSs targeted by 58 human proteins according to the ENCODE ChIP‐Seq experiments (Dunham, 2012) in human embryonic stem cells (H1‐hESC); (4) Gene ontology enrichment using KEGG biological pathways (Kanehisa et al., 2004) as the reference dataset. The latter enrichment analysis was carried out using the *MissMethyl* R package, *gometh* function (Phipson et al., 2016).

## CONFLICT OF INTEREST

The authors declare that they have no conflict of interest.

## AUTHOR CONTRIBUTIONS

Saverio Caini, Domenico Palli, Benedetta Bendinelli, Laura Ottini, Calogero Saieva, and Giovanna Masala involved in conceptualization. Domenico Palli, Benedetta Bendinelli, Giovanna Masala, and Giovanni Fiorito involved in methodology. Giovanni Fiorito, Daniela Ambrogetti, Virginia Valentini, and Piera Rizzolo involved in formal analysis and investigation. Saverio Caini and Giovanni Fiorito involved in writing‐original draft preparation. Domenico Palli, Benedetta Bendinelli, Laura Ottini, Daniela Ambrogetti, Calogero Saieva, Giovanna Masala, Virginia Valentini, and Piera Rizzolo involved in writing‐review and editing. Domenico Palli, Giovanna Masala, and Laura Ottini involved in funding acquisition. Domenico Palli, Giovanna Masala, Laura Ottini, and Daniela Ambrogetti involved in resources. Domenico Palli, Laura Ottini, and Giovanna Masala involved in supervision.

## Supporting information

TABLE S1. Characteristics of the study sample at baseline (absolute number and percentage for categorical variables, mean and standard deviation for continuous variables); *p‐value: Chi‐Squared test for categorical variables; ANOVA test for continuous variables
TABLE S2. Results of the enrichment analyses using the classification of the UCSC Genome Browser for the relationship with CpG islands, open chromatin state and DNase hypersensitivity. P‐values were computed according to the algorithm implemented in the regioneR R package
TABLE S3. Results of the enrichment analyses using the ENCODE classification for chromatin states in embryonic stem cell (H1‐hESC). P‐values were computed according to the algorithm implemented in the *regioneR* R package
TABLE S4. Results of the enrichment analyses using the ENCODE classification for 58 protein TFBS in embryonic stem cell (H1‐hESC). P‐values were computed according to the algorithm implemented in the *regioneR* R package
TABLE S5. Results of the gene ontology enrichment analyses using the KEGG pathways classification as the reference dataset. P‐values were computed according to the algorithm implemented in the *missMethyl* R package, *gometh* function
FIGURE S1. Bisulphite conversion fluorescence intensities for type I (x‐axis) and type II (y‐axis) Illumina450K BeadChip probes. Chips are represented with different colours; the positions on the chip are represented with different symbols (i.e. empty square indicates row 1 and column 1; filled square indicates row 1 and column 2; empty circle indicates row 2 and column 1, etc.). Red dotted lines indicate the threshold of 10,000 suggested by the Illumina guidelines for the identification of bad quality samples to remove. Samples having bisulphite conversion fluorescence intensities lower than 10,000 for both type I and type II probes were excluded from the analyses
FIGURE S2. Combat normalization of fluorescence intensities. Panels A and B reports fluorescence intensities (methylated and non‐methylated probes respectively) by chip before Combat normalization; Panels C and D reports fluorescence intensities (methylated and non‐methylated probes respectively) by chip after Combat normalization. Wet lab analyses were performed in three times, represented with three colors (red, green, and blue)

## Data Availability

The data that support the findings of this study are available on request from the corresponding author. The data are not publicly available due to privacy or ethical restrictions.
