# Supplementary material for: DNA methylation‐based biomarkers of aging were slowed down in a two‐year diet and physical activity intervention trial: the DAMA study
Source: Aging Cell. 2021 Sep 18;20(10):e13439. doi: 10.1111/acel.13439 (PMC8520727; doi:10.1111/acel.13439)
Supplement: Supplementary file 1 — TABLE S1. Characteristics of the study sample at baseline (absolute number and percentage for categorical variables, mean and standard deviation for continuous variables); *p‐value: Chi‐Squared test for categorical variables; ANOVA test for continuous variables TABLE S2. Results of the enrichment analyses using the classification of the UCSC Genome Browser for the relationship with CpG islands, open chromatin state and DNase hypersensitivity. P‐values were computed according to the algorithm implemented in the regioneR R package TABLE S3. Results of the enrichment analyses using the ENCODE classification for chromatin states in embryonic stem cell (H1‐hESC). P‐values were computed according to the algorithm implemented in the regioneR R package TABLE S4. Results of the enrichment analyses using the ENCODE classification for 58 protein TFBS in embryonic stem cell (H1‐hESC). P‐values were computed according to the algorithm implemented in the regioneR R package TABLE S5. Results of the gene ontology enrichment analyses using the KEGG pathways classification as the reference dataset. P‐values were computed according to the algorithm implemented in the missMethyl R package, gometh function FIGURE S1. Bisulphite conversion fluorescence intensities for type I (x‐axis) and type II (y‐axis) Illumina450K BeadChip probes. Chips are represented with different colours; the positions on the chip are represented with different symbols (i.e. empty square indicates row 1 and column 1; filled square indicates row 1 and column 2; empty circle indicates row 2 and column 1, etc.). Red dotted lines indicate the threshold of 10,000 suggested by the Illumina guidelines for the identification of bad quality samples to remove. Samples having bisulphite conversion fluorescence intensities lower than 10,000 for both type I and type II probes were excluded from the analyses FIGURE S2. Combat normalization of fluorescence intensities. Panels A and B reports fluorescence intensities (methylated [file ACEL-20-e13439-s001.docx]

**Figure S1**. Bisulphite conversion fluorescence intensities for type I (x-axis) and type II (y-axis) Illumina450K BeadChip probes. Chips are represented with different colours; the positions on the chip are represented with different symbols (i.e. empty square indicates row 1 and column 1; filled square indicates row 1 and column 2; empty circle indicates row 2 and column 1, etc.). Red dotted lines indicate the threshold of 10,000 suggested by the Illumina guidelines for the identification of bad quality samples to remove. Samples having bisulphite conversion fluorescence intensities lower than 10,000 for both type I and type II probes were excluded from the analyses.


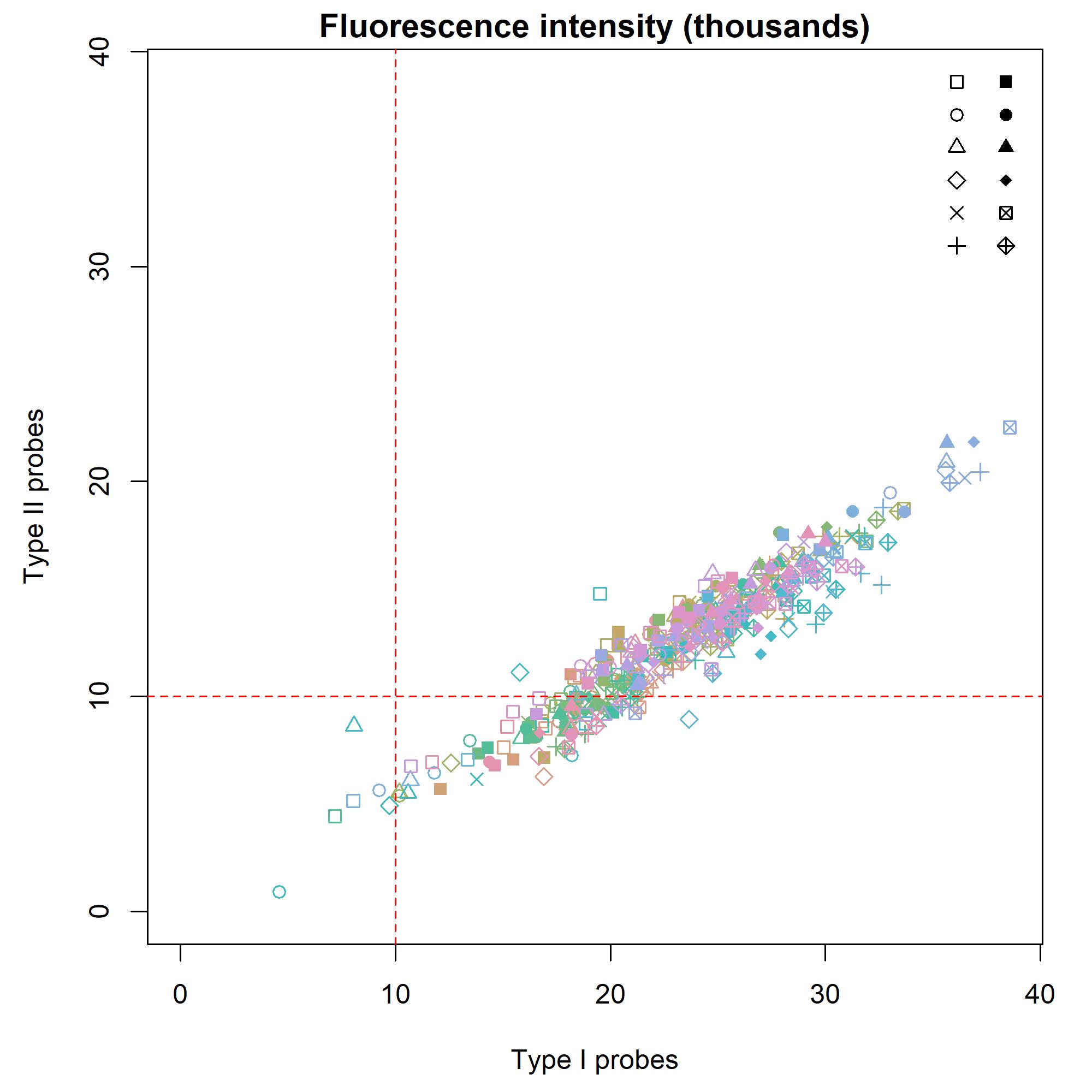


**Figure S2**. Combat normalization of fluorescence intensities. Panels A and B reports fluorescence intensities (methylated and non-methylated probes respectively) by chip before Combat normalization; Panels C and D reports fluorescence intensities (methylated and non-methylated probes respectively) by chip after Combat normalization. Wet lab analyses were performed in three times, represented with three colours (red, green, and blue).


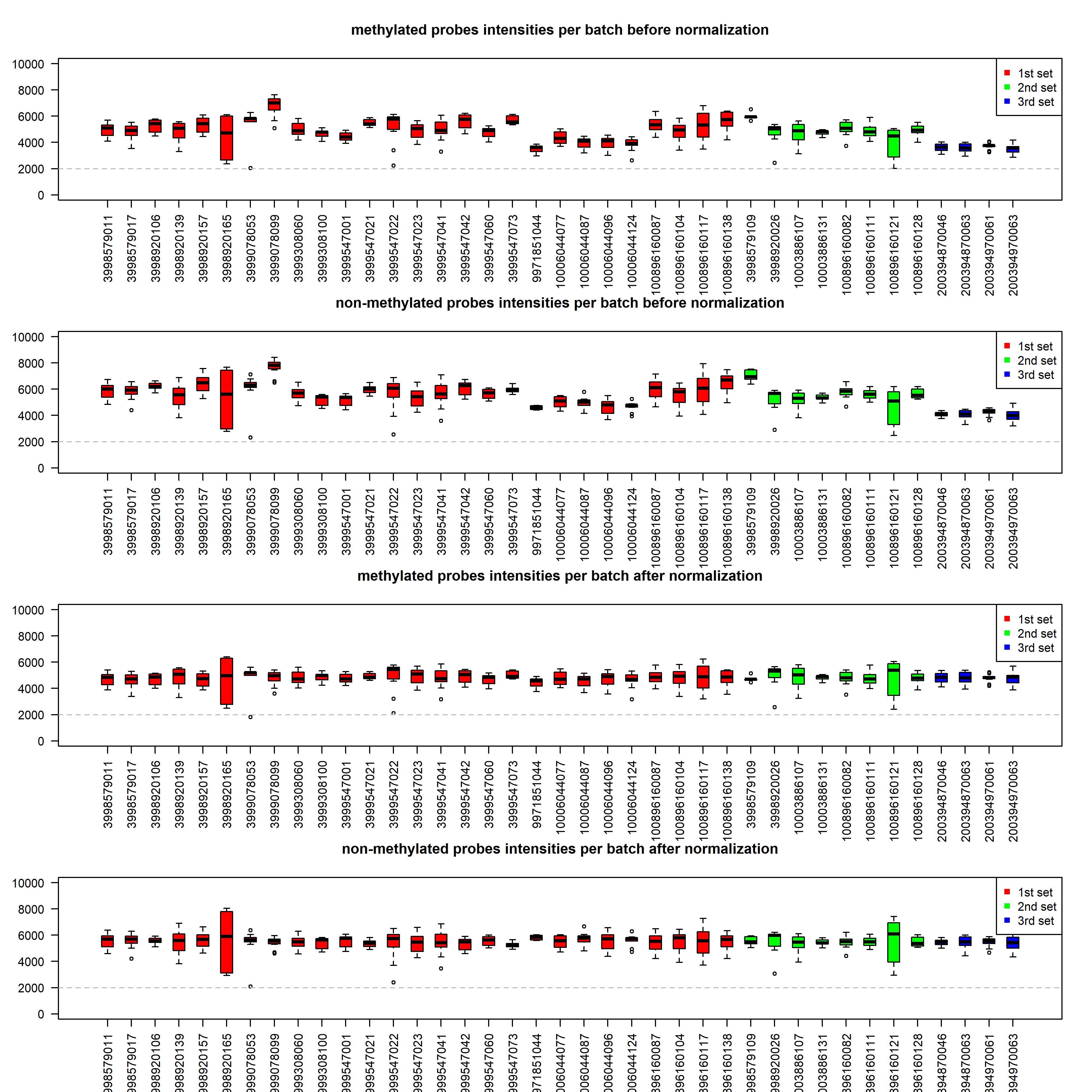


**Table S1**: Characteristics of the study sample at baseline by study arm: *arm 1* = dietary intervention, *arm 2* = PA intervention, *arm 3* = dietary + PA intervention, *arm 4* = control group (absolute number and percentage for categorical variables, mean and standard deviation for continuous variables); *p-value: Chi-Squared test for categorical variables; ANOVA test for continuous variables.

|  | **Control group (arm 4)** | **Dietary intervention group (arm 1)** | **PA intervention group (arm 2)** | **Dietary & PA intervention group (arm 3)** | **p*** |
| --- | --- | --- | --- | --- | --- |
|  | **(n = 56)** | **(n = 56)** | **(n = 55)** | **(n = 52)** |  |
| **Anthropometric and lifestyle characteristics** | | | | | |
| **Age** | 59.15 (5.47) | 58.71 (5.66) | 58.7 (4.74) | 59.36 (4.93) | 0.89 |
| **BMI** |  |  |  |  |  |
| <25 | 34 (60.71%) | 33 (58.93%) | 36 (65.45%) | 34 (65.38%) |  |
| 25-30 | 19 (33.93%) | 20 (35.71%) | 15 (27.27%) | 13 (25.00%) |  |
| >30 | 3 (5.36%) | 3 (5.36%) | 4 (7.27%) | 5 (9.62%) | 0.85 |
| **Smoking** |  |  |  |  |  |
| Never | 35 (62.50%) | 31 (55.36%) | 34 (61.82%) | 27 (51.92%) |  |
| Former | 20 (35.71%) | 25 (44.64%) | 21 (38.18%) | 25 (48.08%) | 0.57 |
| **Education** |  |  |  |  |  |
| None/Primary | 19 (33.93%) | 18 (32.14%) | 15 (27.27%) | 12 (23.08%) |  |
| Secondary | 20 (35.71%) | 24 (42.86%) | 24 (43.64%) | 23 (44.23%) |  |
| University or above | 17 (30.36%) | 14 (25.00%) | 16 (29.09%) | 17 (32.69%) | 0.86 |
| **Physical activity** |  |  |  |  |  |
| Inactive | 14 (25%) | 19 (33.93%) | 14 (25.45%) | 9 (17.31%) |  |
| Mod. inactive | 18 (32.14%) | 18 (32.14%) | 19 (34.55%) | 21 (40.38%) |  |
| Mod. active | 12 (21.43%) | 12 (21.43%) | 16 (29.09%) | 16 (30.77%) |  |
| Active | 12 (21.43%) | 7 (12.50%) | 6 (10.91%) | 6 (11.54%) | 0.53 |
| **Coffee** |  |  |  |  |  |
| <= 3 cups | 30 (53.57%) | 27 (48.21%) | 34 (61.82%) | 24 (46.15%) |  |
| > 3 cups | 26 (46.43%) | 29 (51.79%) | 21 (38.18%) | 28 (53.85%) | 0.36 |
| **Alcohol** |  |  |  |  |  |
| Never | 10 (17.86%) | 8 (14.29%) | 6 (10.91%) | 13 (25.00%) |  |
| <= 1 drink / day | 32 (57.14%) | 36 (64.29%) | 44 (80.00%) | 28 (53.85%) |  |
| > 1 drink / day | 14 (25.00%) | 12 (21.43%) | 5 (9.09%) | 11 (21.15%) | 0.09 |
| **Dietary style*** |  |  |  |  |  |
| MDS > 5 | 30 (53.57%) | 25 (44.64%) | 22 (40.00%) | 22 (42.31%) |  |
| MDS ≤ 5 | 26 (46.43%) | 31 (55.36%) | 33 (60.00%) | 30 (57.69%) | 0.50 |
| **Dietary variables (g/die)** | | | | | |
| **Vegetables** | 201.90 (112.14) | 193.42 (77.63) | 201.83 (109.34) | 212.33 (77.19) | 0.79 |
| **Fruit** | 369.73 (207.18) | 314.63 (161.14) | 317.99 (151.54) | 329.15 (142.42) | 0.28 |
| **Red meat** | 53.83 (33.29) | 53.70 (32.34) | 60.87 (43.49) | 54.29 (33.84) | 0.67 |
| **Processed meat** | 15.85 (14.08) | 17.72 (14.33) | 16.32 (13.91) | 19.41 (15.10) | 0.57 |
| **Poultry** | 25.67 (20.75) | 23.99 (16.84) | 25.69 (21.94) | 27.28 (18.94) | 0.86 |
| **Fish** | 40.41 (23.24) | 40.24 (26.88) | 43.37 (29.68) | 45.38 (29.95) | 0.73 |
| **Dairy products** | 264.96 (171.32) | 223.72 (127.7) | 238.89 (116.46) | 251.98 (192.22) | 0.53 |
| **Kcal** | 2074.54 (590.26) | 1926.12 (502.91) | 2074.12 (645.44) | 2248.13 (764.75) | 0.07 |
| **WBC estimated (Houseman method)** | | | | | |
| **CD8T** | 0.16 (0.05) | 0.16 (0.05) | 0.15 (0.05) | 0.15 (0.05) | 0.80 |
| **CD4T** | 0.18 (0.06) | 0.16 (0.05) | 0.17 (0.06) | 0.17 (0.06) | 0.69 |
| **NK** | 0.02 (0.03) | 0.01 (0.02) | 0.02 (0.04) | 0.02 (0.03) | 0.37 |
| **B cell** | 0.01 (0.01) | 0.01 (0.01) | 0.01 (0.02) | 0.01 (0.01) | 0.83 |
| **Monocytes** | 0.03 (0.02) | 0.03 (0.02) | 0.02 (0.02) | 0.03 (0.02) | 0.74 |
| **Granulocytes** | 0.47 (0.08) | 0.49 (0.07) | 0.49 (0.09) | 0.48 (0.09) | 0.59 |
| **Epigenetic clock measures** | | | | | |
| **DNAmGrimAA** | -0.18 (2.16) | 0.12 (2.3) | -0.02 (2.66) | 0.24 (2.65) | 0.83 |
| **EML** | 6.25 (0.98) | 6.64 (0.94) | 6.09 (0.90) | 6.56 (1.19) | 0.01 |

* Mediterranean Diet Score (MDS) computed as described elsewhere (Fasanelli, 2019).

**Table S2**: Results of the enrichment analyses using the classification of the UCSC Genome Browser for the relationship with CpG islands, open chromatin state and DNase hypersensitivity. P-values were computed according to the algorithm implemented in the regioneR R package.

| **Relation to CpG island according to UCSC Genome Browser** | **Permutation based p-value for enrichment** |
| --- | --- |
| Non CpG Island | 0.02 |
| Shelves | 0.25 |
| DNase hypersensitivity evidence | 0.54 |
| CpG Island | 1.00 |
| Open chromatin evidence | 1.00 |
| Shores | 1.00 |

**Table S3**: Results of the enrichment analyses using the ENCODE classification for chromatin states in embryonic stem cell (H1-hESC). P-values were computed according to the algorithm implemented in the *regioneR* R package.

| **Chromatin state according to ENCODE ChIP-Seq** | **Permutation based p-value for enrichment** |
| --- | --- |
| Heterochromatin / Low signal / CNV | < 0.0001 |
| Inactive / Poised promoter | 0.10 |
| Polycomb Repressed | 0.22 |
| Transcriptional elongation / Transition | 1.00 |
| Weak Transcribed | 1.00 |
| Active promoter | 1.00 |
| Weak promoter | 1.00 |
| Strong enhancer | 1.00 |
| Weak / Poised enhancer | 1.00 |
| Insulator | 1.00 |
| Non regulatory elements | 1.00 |

**Table S4**: Results of the enrichment analyses using the ENCODE classification for 58 protein TFBS in embryonic stem cell (H1-hESC). P-values were computed according to the algorithm implemented in the *regioneR* R package.

| **Transcription Factor** | **Cell** | **Lab** | **Treatment** | **Permutation based p-value for enrichment** |
| --- | --- | --- | --- | --- |
| EZH2 | H1-hESC | Broad | None | 0.001 |
| SUZ12 | H1-hESC | USC | None | 0.006 |
| CtBP2 | H1-hESC | USC | None | 0.22 |
| CHD1 | H1-hESC | Broad | None | 0.25 |
| NRSF | H1-hESC | HudsonAlpha | None | 0.43 |
| BCL11A | H1-hESC | HudsonAlpha | None | 1.00 |
| c-Myc | H1-hESC | UT-A | None | 1.00 |
| POU5F1 | H1-hESC | HudsonAlpha | None | 1.00 |
| MafK | H1-hESC | Stanford | None | 1.00 |
| RXRA | H1-hESC | HudsonAlpha | None | 1.00 |
| NANOG | H1-hESC | HudsonAlpha | None | 1.00 |
| CHD1 | H1-hESC | Stanford | None | 1.00 |
| c-Jun | H1-hESC | Stanford | None | 1.00 |
| RFX5 | H1-hESC | Stanford | None | 1.00 |
| HDAC2 | H1-hESC | HudsonAlpha | None | 1.00 |
| FOSL1 | H1-hESC | HudsonAlpha | None | 1.00 |
| TCF12 | H1-hESC | Stanford | None | 1.00 |
| CEBPB | H1-hESC | HudsonAlpha | None | 1.00 |
| TEAD4 | H1-hESC | USC | None | 1.00 |
| Max | H1-hESC | UT-A | None | 1.00 |
| CTCF | H1-hESC | Stanford | None | 1.00 |
| Rad21 | H1-hESC | Stanford | None | 1.00 |
| Rad21 | H1-hESC | HudsonAlpha | None | 1.00 |
| BRCA1 | H1-hESC | Stanford | None | 1.00 |
| CTCF | H1-hESC | Broad | None | 1.00 |
| CTCF | H1-hESC | Stanford | None | 1.00 |
| USF2 | H1-hESC | Stanford | None | 1.00 |
| SP2 | H1-hESC | HudsonAlpha | None | 1.00 |
| JARID1A | H1-hESC | Broad | None | 1.00 |
| SIX5 | H1-hESC | HudsonAlpha | None | 1.00 |
| SRF | H1-hESC | HudsonAlpha | None | 1.00 |
| USF-1 | H1-hESC | HudsonAlpha | None | 1.00 |
| ATF2 | H1-hESC | HudsonAlpha | None | 1.00 |
| JunD | H1-hESC | Stanford | None | 1.00 |
| ATF3 | H1-hESC | HudsonAlpha | None | 1.00 |
| Bach1 | H1-hESC | Stanford | None | 1.00 |
| Nrf1 | H1-hESC | Stanford | None | 1.00 |
| c-Myc | H1-hESC | Stanford | None | 1.00 |
| JunD | H1-hESC | HudsonAlpha | None | 1.00 |
| GABP | H1-hESC | HudsonAlpha | None | 1.00 |
| GTF2F1 | H1-hESC | Stanford | None | 1.00 |
| p300 | H1-hESC | HudsonAlpha | None | 1.00 |
| Egr-1 | H1-hESC | HudsonAlpha | None | 1.00 |
| Mxi1 | H1-hESC | Stanford | None | 1.00 |
| CHD2 | H1-hESC | Stanford | None | 1.00 |
| Znf143 | H1-hESC | Stanford | None | 1.00 |
| Sin3Ak-20 | H1-hESC | HudsonAlpha | None | 1.00 |
| RBBP5 | H1-hESC | Broad | None | 1.00 |
| YY1 | H1-hESC | HudsonAlpha | None | 1.00 |
| SP1 | H1-hESC | HudsonAlpha | None | 1.00 |
| SP4 | H1-hESC | HudsonAlpha | None | 1.00 |
| TAF7 | H1-hESC | HudsonAlpha | None | 1.00 |
| TBP | H1-hESC | Stanford | None | 1.00 |
| Pol2 | H1-hESC | HudsonAlpha | None | 1.00 |
| SIN3A | H1-hESC | Stanford | None | 1.00 |
| Pol2 | H1-hESC | UT-A | None | 1.00 |
| TAF1 | H1-hESC | HudsonAlpha | None | 1.00 |
| Pol2 | H1-hESC | HudsonAlpha | None | 1.00 |

**Table S5**: Results of the gene ontology enrichment analyses using the KEGG pathways classification as the reference dataset. P-values were computed according to the algorithm implemented in the *missMethyl* R package, *gometh* function.

| **KEGG pathway name** | **Description** | **p** | **FDR p** |
| --- | --- | --- | --- |
| path:hsa05205 | Proteoglycans in cancer | 0.0004 | 0.046 |
| path:hsa05224 | Breast cancer | 0.0006 | 0.046 |
| path:hsa04310 | Wnt signaling pathway | 0.0007 | 0.046 |
| path:hsa04713 | Circadian entrainment | 0.0008 | 0.046 |
| path:hsa04024 | cAMP signaling pathway | 0.0008 | 0.046 |
| path:hsa04390 | Hippo signaling pathway | 0.0009 | 0.046 |
| path:hsa04020 | Calcium signaling pathway | 0.001 | 0.046 |
| path:hsa05412 | Arrhythmogenic right ventricular cardiomyopathy | 0.002 | 0.07 |
| path:hsa05226 | Gastric cancer | 0.002 | 0.07 |
| path:hsa04724 | Glutamatergic synapse | 0.002 | 0.07 |
| path:hsa05414 | Dilated cardiomyopathy | 0.007 | 0.22 |
| path:hsa05218 | Melanoma | 0.008 | 0.22 |
| path:hsa05410 | Hypertrophic cardiomyopathy | 0.01 | 0.25 |
| path:hsa04934 | Cushing syndrome | 0.01 | 0.25 |
| path:hsa04080 | Neuroactive ligand-receptor interaction | 0.01 | 0.26 |
| path:hsa04920 | Adipocytokine signaling pathway | 0.02 | 0.35 |
| path:hsa04725 | Cholinergic synapse | 0.02 | 0.41 |
| path:hsa04510 | Focal adhesion | 0.02 | 0.41 |
| path:hsa04012 | ErbB signaling pathway | 0.03 | 0.45 |
| path:hsa04924 | Renin secretion | 0.03 | 0.45 |
| path:hsa04720 | Long-term potentiation | 0.03 | 0.45 |
| path:hsa04392 | Hippo signaling pathway - multiple species | 0.03 | 0.45 |
| path:hsa00061 | Fatty acid biosynthesis | 0.04 | 0.53 |
| path:hsa04810 | Regulation of actin cytoskeleton | 0.04 | 0.59 |
| path:hsa04022 | cGMP-PKG signaling pathway | 0.05 | 0.59 |
| path:hsa05033 | Nicotine addiction | 0.05 | 0.59 |
| path:hsa05032 | Morphine addiction | 0.06 | 0.72 |
| path:hsa05217 | Basal cell carcinoma | 0.06 | 0.72 |
| path:hsa04970 | Salivary secretion | 0.06 | 0.72 |
| path:hsa04730 | Long-term depression | 0.06 | 0.73 |
| path:hsa04072 | Phospholipase D signaling pathway | 0.07 | 0.73 |
| path:hsa04921 | Oxytocin signaling pathway | 0.07 | 0.76 |
| path:hsa05210 | Colorectal cancer | 0.08 | 0.80 |
| path:hsa04931 | Insulin resistance | 0.09 | 0.89 |
| path:hsa03420 | Nucleotide excision repair | 0.09 | 0.91 |
| path:hsa04742 | Taste transduction | 0.10 | 0.91 |
| path:hsa04723 | Retrograde endocannabinoid signaling | 0.10 | 0.91 |
| path:hsa04010 | MAPK signaling pathway | 0.10 | 0.91 |
| path:hsa04360 | Axon guidance | 0.11 | 0.91 |
| path:hsa04727 | GABAergic synapse | 0.11 | 0.91 |
| path:hsa04371 | Apelin signaling pathway | 0.12 | 0.91 |
| path:hsa01212 | Fatty acid metabolism | 0.12 | 0.91 |
| path:hsa00120 | Primary bile acid biosynthesis | 0.12 | 0.91 |
| path:hsa04014 | Ras signaling pathway | 0.12 | 0.94 |
| path:hsa04151 | PI3K-Akt signaling pathway | 0.13 | 0.94 |
| path:hsa05200 | Pathways in cancer | 0.13 | 0.94 |
| path:hsa01522 | Endocrine resistance | 0.13 | 0.94 |
| path:hsa04540 | Gap junction | 0.13 | 0.94 |
| path:hsa05225 | Hepatocellular carcinoma | 0.14 | 0.97 |
| path:hsa04015 | Rap1 signaling pathway | 0.15 | 1.00 |
| path:hsa04923 | Regulation of lipolysis in adipocytes | 0.16 | 1.00 |
| path:hsa00270 | Cysteine and methionine metabolism | 0.16 | 1.00 |
| path:hsa04512 | ECM-receptor interaction | 0.16 | 1.00 |
| path:hsa04926 | Relaxin signaling pathway | 0.17 | 1.00 |
| path:hsa04911 | Insulin secretion | 0.17 | 1.00 |
| path:hsa05220 | Chronic myeloid leukemia | 0.17 | 1.00 |
| path:hsa05211 | Renal cell carcinoma | 0.18 | 1.00 |
| path:hsa04261 | Adrenergic signaling in cardiomyocytes | 0.19 | 1.00 |
| path:hsa04611 | Platelet activation | 0.19 | 1.00 |
| path:hsa05231 | Choline metabolism in cancer | 0.19 | 1.00 |
| path:hsa05215 | Prostate cancer | 0.19 | 1.00 |
| path:hsa04150 | mTOR signaling pathway | 0.19 | 1.00 |
| path:hsa00250 | Alanine, aspartate and glutamate metabolism | 0.20 | 1.00 |
| path:hsa04340 | Hedgehog signaling pathway | 0.20 | 1.00 |
| path:hsa05202 | Transcriptional misregulation in cancer | 0.21 | 1.00 |
| path:hsa04916 | Melanogenesis | 0.21 | 1.00 |
| path:hsa04974 | Protein digestion and absorption | 0.22 | 1.00 |
| path:hsa05100 | Bacterial invasion of epithelial cells | 0.22 | 1.00 |
| path:hsa05212 | Pancreatic cancer | 0.22 | 1.00 |
| path:hsa04260 | Cardiac muscle contraction | 0.23 | 1.00 |
| path:hsa00532 | Glycosaminoglycan biosynthesis - chondroitin sulfate / dermatan sulfate | 0.23 | 1.00 |
| path:hsa03030 | DNA replication | 0.24 | 1.00 |
| path:hsa01521 | EGFR tyrosine kinase inhibitor resistance | 0.24 | 1.00 |
| path:hsa05222 | Small cell lung cancer | 0.25 | 1.00 |
| path:hsa00472 | D-Arginine and D-ornithine metabolism | 0.26 | 1.00 |
| path:hsa04136 | Autophagy - other | 0.26 | 1.00 |
| path:hsa04270 | Vascular smooth muscle contraction | 0.26 | 1.00 |
| path:hsa04152 | AMPK signaling pathway | 0.26 | 1.00 |
| path:hsa04930 | Type II diabetes mellitus | 0.27 | 1.00 |
| path:hsa05213 | Endometrial cancer | 0.27 | 1.00 |
| path:hsa01040 | Biosynthesis of unsaturated fatty acids | 0.27 | 1.00 |
| path:hsa04950 | Maturity onset diabetes of the young | 0.27 | 1.00 |
| path:hsa05017 | Spinocerebellar ataxia | 0.27 | 1.00 |
| path:hsa04910 | Insulin signaling pathway | 0.27 | 1.00 |
| path:hsa01210 | 2-Oxocarboxylic acid metabolism | 0.27 | 1.00 |
| path:hsa05165 | Human papillomavirus infection | 0.27 | 1.00 |
| path:hsa04728 | Dopaminergic synapse | 0.28 | 1.00 |
| path:hsa04550 | Signaling pathways regulating pluripotency of stem cells | 0.28 | 1.00 |
| path:hsa00604 | Glycosphingolipid biosynthesis - ganglio series | 0.29 | 1.00 |
| path:hsa04913 | Ovarian steroidogenesis | 0.29 | 1.00 |
| path:hsa04216 | Ferroptosis | 0.30 | 1.00 |
| path:hsa04666 | Fc gamma R-mediated phagocytosis | 0.31 | 1.00 |
| path:hsa05214 | Glioma | 0.31 | 1.00 |
| path:hsa00760 | Nicotinate and nicotinamide metabolism | 0.31 | 1.00 |
| path:hsa05135 | Yersinia infection | 0.31 | 1.00 |
| path:hsa04750 | Inflammatory mediator regulation of TRP channels | 0.31 | 1.00 |
| path:hsa04370 | VEGF signaling pathway | 0.32 | 1.00 |
| path:hsa05030 | Cocaine addiction | 0.32 | 1.00 |
| path:hsa05031 | Amphetamine addiction | 0.32 | 1.00 |
| path:hsa00360 | Phenylalanine metabolism | 0.32 | 1.00 |
| path:hsa00220 | Arginine biosynthesis | 0.33 | 1.00 |
| path:hsa00400 | Phenylalanine, tyrosine and tryptophan biosynthesis | 0.33 | 1.00 |
| path:hsa00430 | Taurine and hypotaurine metabolism | 0.33 | 1.00 |
| path:hsa05134 | Legionellosis | 0.34 | 1.00 |
| path:hsa04530 | Tight junction | 0.34 | 1.00 |
| path:hsa04722 | Neurotrophin signaling pathway | 0.34 | 1.00 |
| path:hsa04211 | Longevity regulating pathway | 0.34 | 1.00 |
| path:hsa04213 | Longevity regulating pathway - multiple species | 0.35 | 1.00 |
| path:hsa00410 | beta-Alanine metabolism | 0.35 | 1.00 |
| path:hsa05221 | Acute myeloid leukemia | 0.36 | 1.00 |
| path:hsa00130 | Ubiquinone and other terpenoid-quinone biosynthesis | 0.37 | 1.00 |
| path:hsa04932 | Non-alcoholic fatty liver disease | 0.37 | 1.00 |
| path:hsa04721 | Synaptic vesicle cycle | 0.37 | 1.00 |
| path:hsa04929 | GnRH secretion | 0.37 | 1.00 |
| path:hsa01230 | Biosynthesis of amino acids | 0.38 | 1.00 |
| path:hsa00564 | Glycerophospholipid metabolism | 0.39 | 1.00 |
| path:hsa04972 | Pancreatic secretion | 0.39 | 1.00 |
| path:hsa03430 | Mismatch repair | 0.40 | 1.00 |
| path:hsa04977 | Vitamin digestion and absorption | 0.41 | 1.00 |
| path:hsa05010 | Alzheimer disease | 0.41 | 1.00 |
| path:hsa04971 | Gastric acid secretion | 0.42 | 1.00 |
| path:hsa05120 | Epithelial cell signaling in Helicobacter pylori infection | 0.42 | 1.00 |
| path:hsa05216 | Thyroid cancer | 0.43 | 1.00 |
| path:hsa00750 | Vitamin B6 metabolism | 0.44 | 1.00 |
| path:hsa04514 | Cell adhesion molecules | 0.44 | 1.00 |
| path:hsa04710 | Circadian rhythm | 0.45 | 1.00 |
| path:hsa00020 | Citrate cycle (TCA cycle) | 0.45 | 1.00 |
| path:hsa04925 | Aldosterone synthesis and secretion | 0.45 | 1.00 |
| path:hsa00513 | Various types of N-glycan biosynthesis | 0.45 | 1.00 |
| path:hsa00280 | Valine, leucine and isoleucine degradation | 0.46 | 1.00 |
| path:hsa04140 | Autophagy - animal | 0.46 | 1.00 |
| path:hsa04350 | TGF-beta signaling pathway | 0.46 | 1.00 |
| path:hsa04068 | FoxO signaling pathway | 0.46 | 1.00 |
| path:hsa04927 | Cortisol synthesis and secretion | 0.46 | 1.00 |
| path:hsa00310 | Lysine degradation | 0.46 | 1.00 |
| path:hsa02010 | ABC transporters | 0.46 | 1.00 |
| path:hsa00062 | Fatty acid elongation | 0.46 | 1.00 |
| path:hsa00561 | Glycerolipid metabolism | 0.48 | 1.00 |
| path:hsa05146 | Amoebiasis | 0.49 | 1.00 |
| path:hsa04966 | Collecting duct acid secretion | 0.49 | 1.00 |
| path:hsa05310 | Asthma | 0.50 | 1.00 |
| path:hsa05206 | MicroRNAs in cancer | 0.50 | 1.00 |
| path:hsa04062 | Chemokine signaling pathway | 0.50 | 1.00 |
| path:hsa04976 | Bile secretion | 0.51 | 1.00 |
| path:hsa05321 | Inflammatory bowel disease | 0.52 | 1.00 |
| path:hsa04640 | Hematopoietic cell lineage | 0.53 | 1.00 |
| path:hsa04218 | Cellular senescence | 0.53 | 1.00 |
| path:hsa04979 | Cholesterol metabolism | 0.53 | 1.00 |
| path:hsa03018 | RNA degradation | 0.53 | 1.00 |
| path:hsa00440 | Phosphonate and phosphinate metabolism | 0.53 | 1.00 |
| path:hsa05223 | Non-small cell lung cancer | 0.54 | 1.00 |
| path:hsa04960 | Aldosterone-regulated sodium reabsorption | 0.54 | 1.00 |
| path:hsa00770 | Pantothenate and CoA biosynthesis | 0.54 | 1.00 |
| path:hsa04928 | Parathyroid hormone synthesis, secretion and action | 0.55 | 1.00 |
| path:hsa00290 | Valine, leucine and isoleucine biosynthesis | 0.55 | 1.00 |
| path:hsa05219 | Bladder cancer | 0.55 | 1.00 |
| path:hsa00524 | Neomycin, kanamycin and gentamicin biosynthesis | 0.55 | 1.00 |
| path:hsa04914 | Progesterone-mediated oocyte maturation | 0.55 | 1.00 |
| path:hsa05163 | Human cytomegalovirus infection | 0.56 | 1.00 |
| path:hsa04922 | Glucagon signaling pathway | 0.57 | 1.00 |
| path:hsa04610 | Complement and coagulation cascades | 0.57 | 1.00 |
| path:hsa00330 | Arginine and proline metabolism | 0.57 | 1.00 |
| path:hsa00350 | Tyrosine metabolism | 0.59 | 1.00 |
| path:hsa04625 | C-type lectin receptor signaling pathway | 0.59 | 1.00 |
| path:hsa04917 | Prolactin signaling pathway | 0.59 | 1.00 |
| path:hsa04726 | Serotonergic synapse | 0.59 | 1.00 |
| path:hsa03410 | Base excision repair | 0.60 | 1.00 |
| path:hsa04919 | Thyroid hormone signaling pathway | 0.60 | 1.00 |
| path:hsa04744 | Phototransduction | 0.61 | 1.00 |
| path:hsa04961 | Endocrine and other factor-regulated calcium reabsorption | 0.62 | 1.00 |
| path:hsa00900 | Terpenoid backbone biosynthesis | 0.63 | 1.00 |
| path:hsa04935 | Growth hormone synthesis, secretion and action | 0.64 | 1.00 |
| path:hsa04964 | Proximal tubule bicarbonate reclamation | 0.65 | 1.00 |
| path:hsa00512 | Mucin type O-glycan biosynthesis | 0.66 | 1.00 |
| path:hsa00563 | Glycosylphosphatidylinositol (GPI)-anchor biosynthesis | 0.66 | 1.00 |
| path:hsa04612 | Antigen processing and presentation | 0.67 | 1.00 |
| path:hsa05416 | Viral myocarditis | 0.67 | 1.00 |
| path:hsa03320 | PPAR signaling pathway | 0.68 | 1.00 |
| path:hsa04115 | p53 signaling pathway | 0.68 | 1.00 |
| path:hsa00140 | Steroid hormone biosynthesis | 0.69 | 1.00 |
| path:hsa04146 | Peroxisome | 0.69 | 1.00 |
| path:hsa05161 | Hepatitis B | 0.70 | 1.00 |
| path:hsa05230 | Central carbon metabolism in cancer | 0.70 | 1.00 |
| path:hsa04670 | Leukocyte transendothelial migration | 0.70 | 1.00 |
| path:hsa05110 | Vibrio cholerae infection | 0.72 | 1.00 |
| path:hsa00592 | alpha-Linolenic acid metabolism | 0.72 | 1.00 |
| path:hsa00260 | Glycine, serine and threonine metabolism | 0.73 | 1.00 |
| path:hsa04137 | Mitophagy - animal | 0.74 | 1.00 |
| path:hsa04520 | Adherens junction | 0.74 | 1.00 |
| path:hsa04915 | Estrogen signaling pathway | 0.74 | 1.00 |
| path:hsa00340 | Histidine metabolism | 0.75 | 1.00 |
| path:hsa00534 | Glycosaminoglycan biosynthesis - heparan sulfate / heparin | 0.75 | 1.00 |
| path:hsa03013 | RNA transport | 0.75 | 1.00 |
| path:hsa00531 | Glycosaminoglycan degradation | 0.75 | 1.00 |
| path:hsa05418 | Fluid shear stress and atherosclerosis | 0.75 | 1.00 |
| path:hsa04630 | JAK-STAT signaling pathway | 0.75 | 1.00 |
| path:hsa00533 | Glycosaminoglycan biosynthesis - keratan sulfate | 0.76 | 1.00 |
| path:hsa00510 | N-Glycan biosynthesis | 0.77 | 1.00 |
| path:hsa05166 | Human T-cell leukemia virus 1 infection | 0.77 | 1.00 |
| path:hsa05145 | Toxoplasmosis | 0.77 | 1.00 |
| path:hsa00603 | Glycosphingolipid biosynthesis - globo and isoglobo series | 0.77 | 1.00 |
| path:hsa00670 | One carbon pool by folate | 0.77 | 1.00 |
| path:hsa04622 | RIG-I-like receptor signaling pathway | 0.77 | 1.00 |
| path:hsa04114 | Oocyte meiosis | 0.77 | 1.00 |
| path:hsa05144 | Malaria | 0.77 | 1.00 |
| path:hsa00790 | Folate biosynthesis | 0.78 | 1.00 |
| path:hsa00640 | Propanoate metabolism | 0.78 | 1.00 |
| path:hsa04662 | B cell receptor signaling pathway | 0.78 | 1.00 |
| path:hsa04614 | Renin-angiotensin system | 0.78 | 1.00 |
| path:hsa04664 | Fc epsilon RI signaling pathway | 0.79 | 1.00 |
| path:hsa00780 | Biotin metabolism | 0.79 | 1.00 |
| path:hsa00040 | Pentose and glucuronate interconversions | 0.79 | 1.00 |
| path:hsa05020 | Prion diseases | 0.79 | 1.00 |
| path:hsa05169 | Epstein-Barr virus infection | 0.80 | 1.00 |
| path:hsa04933 | AGE-RAGE signaling pathway in diabetic complications | 0.80 | 1.00 |
| path:hsa00620 | Pyruvate metabolism | 0.80 | 1.00 |
| path:hsa00100 | Steroid biosynthesis | 0.80 | 1.00 |
| path:hsa04918 | Thyroid hormone synthesis | 0.80 | 1.00 |
| path:hsa03040 | Spliceosome | 0.80 | 1.00 |
| path:hsa04215 | Apoptosis - multiple species | 0.81 | 1.00 |
| path:hsa00190 | Oxidative phosphorylation | 0.81 | 1.00 |
| path:hsa04912 | GnRH signaling pathway | 0.81 | 1.00 |
| path:hsa00650 | Butanoate metabolism | 0.81 | 1.00 |
| path:hsa05034 | Alcoholism | 0.81 | 1.00 |
| path:hsa05323 | Rheumatoid arthritis | 0.81 | 1.00 |
| path:hsa03050 | Proteasome | 0.82 | 1.00 |
| path:hsa04714 | Thermogenesis | 0.82 | 1.00 |
| path:hsa01523 | Antifolate resistance | 0.82 | 1.00 |
| path:hsa04066 | HIF-1 signaling pathway | 0.82 | 1.00 |
| path:hsa00600 | Sphingolipid metabolism | 0.83 | 1.00 |
| path:hsa04660 | T cell receptor signaling pathway | 0.83 | 1.00 |
| path:hsa04672 | Intestinal immune network for IgA production | 0.83 | 1.00 |
| path:hsa01200 | Carbon metabolism | 0.83 | 1.00 |
| path:hsa04330 | Notch signaling pathway | 0.84 | 1.00 |
| path:hsa04145 | Phagosome | 0.84 | 1.00 |
| path:hsa05322 | Systemic lupus erythematosus | 0.84 | 1.00 |
| path:hsa00071 | Fatty acid degradation | 0.84 | 1.00 |
| path:hsa04380 | Osteoclast differentiation | 0.85 | 1.00 |
| path:hsa04061 | Viral protein interaction with cytokine and cytokine receptor | 0.85 | 1.00 |
| path:hsa04110 | Cell cycle | 0.85 | 1.00 |
| path:hsa04668 | TNF signaling pathway | 0.85 | 1.00 |
| path:hsa04210 | Apoptosis | 0.85 | 1.00 |
| path:hsa00740 | Riboflavin metabolism | 0.85 | 1.00 |
| path:hsa05130 | Pathogenic Escherichia coli infection | 0.86 | 1.00 |
| path:hsa00601 | Glycosphingolipid biosynthesis - lacto and neolacto series | 0.86 | 1.00 |
| path:hsa05170 | Human immunodeficiency virus 1 infection | 0.87 | 1.00 |
| path:hsa03450 | Non-homologous end-joining | 0.87 | 1.00 |
| path:hsa04659 | Th17 cell differentiation | 0.87 | 1.00 |
| path:hsa04962 | Vasopressin-regulated water reabsorption | 0.87 | 1.00 |
| path:hsa05235 | PD-L1 expression and PD-1 checkpoint pathway in cancer | 0.87 | 1.00 |
| path:hsa04144 | Endocytosis | 0.88 | 1.00 |
| path:hsa05150 | Staphylococcus aureus infection | 0.88 | 1.00 |
| path:hsa04620 | Toll-like receptor signaling pathway | 0.88 | 1.00 |
| path:hsa05152 | Tuberculosis | 0.89 | 1.00 |
| path:hsa04973 | Carbohydrate digestion and absorption | 0.89 | 1.00 |
| path:hsa05203 | Viral carcinogenesis | 0.89 | 1.00 |
| path:hsa00053 | Ascorbate and aldarate metabolism | 0.89 | 1.00 |
| path:hsa05167 | Kaposi sarcoma-associated herpesvirus infection | 0.89 | 1.00 |
| path:hsa04141 | Protein processing in endoplasmic reticulum | 0.89 | 1.00 |
| path:hsa03015 | mRNA surveillance pathway | 0.90 | 1.00 |
| path:hsa00730 | Thiamine metabolism | 0.90 | 1.00 |
| path:hsa04978 | Mineral absorption | 0.90 | 1.00 |
| path:hsa04658 | Th1 and Th2 cell differentiation | 0.90 | 1.00 |
| path:hsa00030 | Pentose phosphate pathway | 0.91 | 1.00 |
| path:hsa00471 | D-Glutamine and D-glutamate metabolism | 0.91 | 1.00 |
| path:hsa05162 | Measles | 0.91 | 1.00 |
| path:hsa01524 | Platinum drug resistance | 0.91 | 1.00 |
| path:hsa00514 | Other types of O-glycan biosynthesis | 0.91 | 1.00 |
| path:hsa04130 | SNARE interactions in vesicular transport | 0.91 | 1.00 |
| path:hsa05340 | Primary immunodeficiency | 0.92 | 1.00 |
| path:hsa00051 | Fructose and mannose metabolism | 0.92 | 1.00 |
| path:hsa00515 | Mannose type O-glycan biosynthesis | 0.92 | 1.00 |
| path:hsa05142 | Chagas disease | 0.92 | 1.00 |
| path:hsa00010 | Glycolysis / Gluconeogenesis | 0.93 | 1.00 |
| path:hsa00970 | Aminoacyl-tRNA biosynthesis | 0.93 | 1.00 |
| path:hsa05204 | Chemical carcinogenesis | 0.93 | 1.00 |
| path:hsa00630 | Glyoxylate and dicarboxylate metabolism | 0.93 | 1.00 |
| path:hsa03020 | RNA polymerase | 0.93 | 1.00 |
| path:hsa00240 | Pyrimidine metabolism | 0.93 | 1.00 |
| path:hsa05168 | Herpes simplex virus 1 infection | 0.94 | 1.00 |
| path:hsa00860 | Porphyrin and chlorophyll metabolism | 0.94 | 1.00 |
| path:hsa05160 | Hepatitis C | 0.94 | 1.00 |
| path:hsa03460 | Fanconi anemia pathway | 0.95 | 1.00 |
| path:hsa05016 | Huntington disease | 0.95 | 1.00 |
| path:hsa05140 | Leishmaniasis | 0.95 | 1.00 |
| path:hsa00072 | Synthesis and degradation of ketone bodies | 0.95 | 1.00 |
| path:hsa05014 | Amyotrophic lateral sclerosis | 0.95 | 1.00 |
| path:hsa04071 | Sphingolipid signaling pathway | 0.95 | 1.00 |
| path:hsa00591 | Linoleic acid metabolism | 0.95 | 1.00 |
| path:hsa05012 | Parkinson disease | 0.96 | 1.00 |
| path:hsa04120 | Ubiquitin mediated proteolysis | 0.96 | 1.00 |
| path:hsa00380 | Tryptophan metabolism | 0.96 | 1.00 |
| path:hsa04122 | Sulfur relay system | 0.96 | 1.00 |
| path:hsa05132 | Salmonella infection | 0.97 | 1.00 |
| path:hsa00511 | Other glycan degradation | 0.97 | 1.00 |
| path:hsa05143 | African trypanosomiasis | 0.97 | 1.00 |
| path:hsa00500 | Starch and sucrose metabolism | 0.97 | 1.00 |
| path:hsa04142 | Lysosome | 0.97 | 1.00 |
| path:hsa05164 | Influenza A | 0.98 | 1.00 |
| path:hsa05133 | Pertussis | 0.98 | 1.00 |
| path:hsa04217 | Necroptosis | 0.98 | 1.00 |
| path:hsa04940 | Type I diabetes mellitus | 0.98 | 1.00 |
| path:hsa00565 | Ether lipid metabolism | 0.98 | 1.00 |
| path:hsa04064 | NF-kappa B signaling pathway | 0.98 | 1.00 |
| path:hsa00230 | Purine metabolism | 0.98 | 1.00 |
| path:hsa04657 | IL-17 signaling pathway | 0.98 | 1.00 |
| path:hsa04621 | NOD-like receptor signaling pathway | 0.98 | 1.00 |
| path:hsa00830 | Retinol metabolism | 0.98 | 1.00 |
| path:hsa04623 | Cytosolic DNA-sensing pathway | 0.98 | 1.00 |
| path:hsa05320 | Autoimmune thyroid disease | 0.98 | 1.00 |
| path:hsa00480 | Glutathione metabolism | 0.98 | 1.00 |
| path:hsa04060 | Cytokine-cytokine receptor interaction | 0.99 | 1.00 |
| path:hsa03440 | Homologous recombination | 0.99 | 1.00 |
| path:hsa00590 | Arachidonic acid metabolism | 0.99 | 1.00 |
| path:hsa00980 | Metabolism of xenobiotics by cytochrome P450 | 0.99 | 1.00 |
| path:hsa03008 | Ribosome biogenesis in eukaryotes | 0.99 | 1.00 |
| path:hsa03022 | Basal transcription factors | 0.99 | 1.00 |
| path:hsa05330 | Allograft rejection | 0.99 | 1.00 |
| path:hsa00052 | Galactose metabolism | 0.99 | 1.00 |
| path:hsa00910 | Nitrogen metabolism | 0.99 | 1.00 |
| path:hsa04070 | Phosphatidylinositol signaling system | 0.99 | 1.00 |
| path:hsa03060 | Protein export | 0.99 | 1.00 |
| path:hsa05332 | Graft-versus-host disease | 0.99 | 1.00 |
| path:hsa00562 | Inositol phosphate metabolism | 0.99 | 1.00 |
| path:hsa04975 | Fat digestion and absorption | 1.00 | 1.00 |
| path:hsa00520 | Amino sugar and nucleotide sugar metabolism | 1.00 | 1.00 |
| path:hsa04740 | Olfactory transduction | 1.00 | 1.00 |
| path:hsa05131 | Shigellosis | 1.00 | 1.00 |
| path:hsa00983 | Drug metabolism - other enzymes | 1.00 | 1.00 |
| path:hsa00982 | Drug metabolism - cytochrome P450 | 1.00 | 1.00 |
| path:hsa00450 | Selenocompound metabolism | 1.00 | 1.00 |
| path:hsa01100 | Metabolic pathways | 1.00 | 1.00 |
| path:hsa04650 | Natural killer cell mediated cytotoxicity | 1.00 | 1.00 |
| path:hsa03010 | Ribosome | 1.00 | 1.00 |
| path:hsa00232 | Caffeine metabolism | 1.00 | 1.00 |
| path:hsa00785 | Lipoic acid metabolism | 1.00 | 1.00 |
| path:hsa00920 | Sulfur metabolism | 1.00 | 1.00 |
